# Applications of Natural Language Processing and Large Language Models for Social Determinants of Health: Systematic Review

**DOI:** 10.2196/83793

**Published:** 2026-04-28

**Authors:** Swati Rajwal, Avinash Kumar Pandey, Ziyuan Zhang, Yankai Chen, Michael X Liu, Sudeshna Das, Hannah Rogers, Abeed Sarker, Yunyu Xiao

**Affiliations:** 1 Department of Biomedical Informatics, School of Medicine, Emory University, 101 Woodruff Circle, Atlanta, GA, 30322, United States, 1 4704478469; 2 Laney Graduate School, Emory University, Atlanta, GA, United States; 3 Department of Epidemiology, University of California, Berkeley, Berkeley, CA, United States; 4 Department of Population Health Sciences, Cornell University, New York, NY, United States; 5 Woodruff Health Sciences Center Library, Emory University, Atlanta, GA, United States

**Keywords:** social determinants of health, SDOH, natural language processing, large language models, systematic review, PRISMA

## Abstract

**Background:**

Social determinants of health (SDOH) are the social, economic, and environmental conditions that influence health outcomes. SDOH information is often embedded in unstructured text, such as notes in electronic health records and social media posts. Advances in natural language processing (NLP), including emergent large language models (LLMs), offer opportunities to extract, analyze, and interpret SDOH expressions from free text for inclusion in downstream analyses. Existing literature on NLP applications for SDOH is dispersed across disciplines and characterized by methodological heterogeneity and variability in study quality and scope, complicating synthesis and cross-study comparison.

**Objective:**

This study aimed to examine the use of NLP, including LLMs, in SDOH research, and highlight gaps and future research directions.

**Methods:**

We conducted a systematic review following PRISMA (Preferred Reporting Items for Systematic Reviews and Meta-Analyses) guidelines, searching 7 major databases for publications between 2014 and November 2025. We included journal and conference proceedings papers that applied NLP methods to identify, classify, extract, or predict SDOH from text. Three reviewers independently screened studies and extracted data; conflicts were resolved by two senior reviewers. We abstracted study metadata, dataset characteristics, NLP approaches, SDOH domains addressed, and NLP performance metrics. We also conducted risk-of-bias analyses and identified influential studies based on relative citation counts.

**Results:**

142 studies met the inclusion criteria. Nearly two-thirds (89/142, 62.7%) were published between 2023 and 2025, reflecting rapid recent growth. Most studies relied on electronic health records (93/142, 65.5%) and private datasets (81/142, 57.0%), while only 20.4% (29/142) used publicly available data. Commonly studied SDOH domains were housing instability (72/142, 50.7%), employment (65/142, 45.8%), and financial conditions (63/142, 44.4%); structural factors, such as immigration status (5/142, 3.5%), were rarely examined. Of studies that reported evaluation metrics, most focused on classification (26/83, 31.32%) or extraction (38/83, 45.7%), and used cross-sectional designs. Reported model performances were typically strong, with median *F*_1_-scores ranging roughly from 0.75 to 0.85 across model categories. Only 49 studies shared code, and fewer than half clearly described model interpretability or reproducibility practices. LLMs (including encoder-decoder models) appeared in 19.7% (28/142) of studies, highlighting emerging interest but also raising new concerns around transparency and governance.

**Conclusions:**

This review provides a timely synthesis of NLP and LLM applications across the SDOH research spectrum, addressing an important gap in a topic receiving increasing research attention. By comparing task formulations, data sources, and performance patterns, the review clarifies the research readiness of current approaches and reveals critical gaps. Our findings advance the field by highlighting the absence of a unified SDOH framework, uneven availability of public benchmarks, and limited evaluation of real-world deployment. Addressing these gaps through transparent, inclusive dataset development and implementation-focused evaluation is essential for translating NLP advances into equitable, real-world health impact.

## Introduction

Social determinants of health (SDOH) refer to nonmedical factors such as the social, economic, and environmental conditions that shape where people live, work, and age. SDOH are among the most powerful drivers of health outcomes and disparities worldwide [1,2], with influences on health [3] and well-being at the individual and population levels [4]. Contemporary estimates suggest that medical care accounts for only 10%‐20% of the modifiable contributors to healthy outcomes, while SDOH-related factors drive the remaining 80%‐90% [3,5,6]. Consequently, SDOH factors, such as housing instability, food insecurity, and structural racism, have become central to understanding the persistence of disease, quality of life, and mortality across diverse populations [7,8]. Accurately capturing SDOH and incorporating them into health care is crucial for clinicians, health systems, and policymakers aiming to address structural inequities and enhance care delivery. Clinicians, for example, can work with patients in social prescribing based on affordability or the availability of transportation to the relevant pharmacy. Similarly, with an in-depth understanding of structural SDOH, including place-based contextual metrics of economic, educational, health, and environmental conditions, policymakers may effectively guide health policies [9-11]. While health systems increasingly recognize that addressing social needs is essential for value-based care, the mechanisms to systematically identify and act upon these factors are often underdeveloped [12].

Several frameworks have been proposed to characterize SDOH into groups. The Healthy People 2030 initiative [2] groups SDOH into 5 domains: Economic Stability, Education Access and Quality, Health Care Access and Quality, Neighborhood and Built Environment, and Social and Community Context. The World Health Organization conceptualizes SDOH into 2 interacting groups: Structural and Intermediary, with the former having causal priority in influencing health outcomes. Due to their importance in influencing health, SDOH codes were also introduced into the International Classification of Diseases, 10th Revision, Clinical Modification in 2015. These are categorized as Z-codes (Z55-Z65) and are used to document socioeconomic and psychosocial circumstances that affect a person’s health. Yet, the documentation of Z-codes has been low in health systems [13]. Thus, SDOH information is often buried in unstructured text, such as notes in electronic health records (EHRs) and social media postings, where it may be expressed through nuanced or implicit language, limiting information accessibility through conventional methods and requiring the development of customized text mining approaches [14].

Natural language processing (NLP) is a subfield of artificial intelligence and computer science that enables computers to process, understand, interpret, and generate human language in meaningful and useful ways. NLP encompasses a wide range of tasks, including but not limited to text classification, information extraction/entity recognition, text summarization, and language translation. NLP offers a promising tool to systematically analyze vast amounts of unstructured text data, EHRs, social media [15,16], public health reports, and other sources [17]. While biomedical domain NLP has been widely applied to extract clinical concepts such as diagnoses, medications, and procedures, much less work has focused on nonclinical aspects, such as SDOH and their influence on health outcomes.

In the context of SDOH, NLP can assist in parsing and understanding context, co-references, and the relationships between text parts. Unlike manual review (time-consuming, labor-intensive, and prone to human error), NLP enables the rapid and scalable analysis of large datasets with greater accuracy and consistency [18,19] when models are appropriately adapted to the domain and data, although performance may vary across populations and SDOH categories. A common NLP application is the identification and extraction of SDOH factors from EHRs and other text-based data, enabling the systematic classification of social needs, behavioral drivers, and environmental conditions that influence patient outcomes [20-24]. The versatility of NLP is evident in its application across diverse clinical domains, ranging from pediatric populations [25,26] to patients managing chronic conditions such as Alzheimer disease [22] and lower back pain [27]. Beyond traditional medical notes, NLP workflows have been adapted to mine insights from specialized documentation, including clinical social work notes [28] and emergency medical services records [29], as well as nonclinical data sources like social media, where it has been used to assess the impact of external crises (eg, COVID-19) on marginalized communities [30]. Underpinning these diverse applications is the rapid evolution of model architectures; specifically, the shift toward transformer-based models and generative pretrained transformers has significantly enhanced the precision of SDOH extraction from free-text data [31,32].

Despite these technological advancements, the literature remains fragmented in the application of NLP to characterize SDOH, due to disparate disciplines, data modalities, and methodological frameworks. A comprehensive NLP workflow for SDOH analysis necessitates a rigorous pipeline: (1) defining the target SDOH elements or categories; (2) selecting the appropriate NLP modeling strategy (eg, classification vs extraction); (3) curating gold-standard annotated datasets for supervision and validation; and (4) deploying the optimal strategy. Currently, no comprehensive synthesis exists that integrates these myriad applications of NLP and large language models (LLMs) for SDOH. Consequently, there is a critical knowledge gap regarding the most effective approaches to modeling SDOH, limiting the translation of these technical capabilities into standardized public health and clinical practice.

To address this gap, we conducted a systematic review of peer-reviewed studies published between 2014 and 2025. This period is marked by the introduction of Word2Vec [33], transformer models [34] such as BERT [35], and generative models like the generative pretrained transformer series [36]. Our review consolidates evidence from health, informatics, and computer science literature to assess the state of the science in applying NLP to SDOH. We broadly categorize SDOH into individual and structural factors, a simplification of more comprehensive frameworks such as Healthy People 2030 and the World Health Organization Commission on SDOH. Individual-level SDOH refer to personal circumstances such as income, education, employment status, housing conditions, and access to health care, which directly influence an individual’s health outcomes. Structural SDOH encompass the broader systemic and institutional contexts (such as policies, social norms, economic systems, and structural racism) that shape and constrain the distribution of individual-level resources and opportunities [10]. This systematic review has two major objectives: (1) to characterize NLP techniques, including LLMs, used to analyze SDOH in unstructured individual and public data, including annotation, prediction, detection, and classification; and (2) to assess the effectiveness of such techniques or models, identify potential knowledge gaps, and reveal research questions relevant for future studies.

## Methods

### Registration and Protocol

The systematic review was registered in PROSPERO (CRD42024578082), and the protocol was published in JMIR Research Protocol [37]. The review followed the PRISMA (Preferred Reporting Items for Systematic Reviews and Meta-Analyses) guidelines [38], and the search strategy was reported in accordance with PRISMA-S [39]. We conducted a narrative synthesis without meta-analysis following the Synthesis Without Meta-analysis guidelines [40] due to substantial methodological heterogeneity across studies. Formal subgroup, heterogeneity, sensitivity analyses, reporting bias analysis, or certainty of evidence assessment were not conducted as the review did not involve meta-analysis and instead examined differences across studies descriptively. Synthesis focused on identifying patterns and trends in NLP methods, SDOH domains, data sources, and reported performance rather than pooling quantitative estimates. The interdisciplinary review team consisted of a librarian and researchers with expertise in health informatics, public health, and social sciences.

### Ethical Considerations

This work does not involve human participants and does not require approval from the IRB.

### Eligibility Criteria

We included peer-reviewed literature (journal articles and full conference papers) published between January 2014 and November 2025. The selection of this timeframe was deliberate, as it strategically captures the entire modern deep learning era in NLP, from the rise of word embeddings (eg, Word2Vec) around 2014 to the development of the transformer architecture in 2017 and the subsequent explosion of LLMs. Only studies in English were included, although the underlying NLP data could be in any language. Eligible studies met the following specific criteria: (1) addressed a research question involving the design, development, and application of NLP in health data analysis for SDOH; (2) used text-based data; and (3) used NLP techniques or LLMs (open-source or commercial) related to SDOH. Studies using NLP in conjunction with other methods were eligible, provided NLP was a core component. Preprints (arXiv/bioRxiv), forewords, prefaces, table of contents, programs, schedules, indexes, call for papers or participation, lists of reviewers, lists of tutorial abstracts, invited talks, appendices, session information, obituaries, book reviews, newsletters, lists of proceedings, lifetime achievement awards, erratum, systematic reviews, scoping reviews, and notes were excluded.

### Database and Search Strategy

We systematically searched 7 databases: PubMed, Scopus, Web of Science, PsycINFO, Health Source: Nursing/Academic, ACL Anthology, and IEEE Xplore. Searches were conducted independently in each database rather than through a multi-database platform. No study registries, manual browsing of web-based resources, citation chaining, or direct contact with authors or experts were undertaken as part of the search methodology. The search strategies were developed specifically for this review and were not adapted from previous reviews. No additional information sources or search methods were used.

The search spanned publications from January 1, 2014, to November 2, 2025. A health sciences librarian (HR) developed and iteratively refined the search strategy in consultation with the research team, using both controlled vocabulary and free-text terms. The final PubMed search strategy, including filters, is detailed in Textbox 1. Comprehensive search strategies for all information sources are provided in Multimedia Appendix 1.

Textbox 1. **Search query for PubMed. A comprehensive list of all search queries customized for each database is available in Multimedia Appendix 1.**

**Query:**

- (“Natural Language Processing”[Mesh] OR “natural language”[tw] OR NLP[tw] OR “large LM*”[tw] OR LLM[tw] OR LLMs[tw] OR “large language model*”[tw] or ChatGPT*[tw] OR “Chat GPT*”[tw] OR GPT4*[tw] OR GPT-4*[tw] OR GPT3*[tw] OR GPT-3*[tw] OR “Generative Pre-trained Transformer*”[tw] OR LLAMA[tw] OR “Claude 3”[tw] OR Mistral[tw] OR MedPaLM*[tw] OR Med-PaLM*[tw] OR “text mining”[tw] OR “text process*”[tw] OR “information retrieval”[tw] OR “information extract*”[tw]) AND (“Social Determinants of Health”[Mesh] OR SDOH[tw] OR SDH[tw] OR SBDH*[tw] OR “determinants of health”[tw] OR “health determina*”[tw] OR “life events”[tw] OR “social determinant*”[tw] OR “socioeconomic determinant*”[tw] OR “socioeconomic factor*”[tw] OR “social determinate*”[tw] OR “social factor*”[tw] OR “social need*”[tw] OR “social prescribing”[tw] OR “social determining factor*”[tw] OR “social risk*”[tw])

**Filters:**

- Language: English
- Years: 2014‐2025
- Exclude: Preprints

### Selection Process

Three reviewers (SR, ZZ, and YC) independently screened each study for eligibility by marking it as a “yes” (for inclusion), “no” (for exclusion), or “maybe” (in case of uncertainty about relevance) on the Covidence platform [41]. Two senior reviewers (AS and YX) resolved potential discrepancies during any screening step. Blinded voting ensured that reviewers did not view others’ decisions during the screening process. The reviewers retrieved eligible studies for second-stage review (full-text) using the same inclusion criteria and removed those that did not meet them. The final set of studies to include was approved by consensus of all reviewers.

### Data Extraction

Before formal data extraction, 3 reviewers (SR, ZZ, and YC) piloted a structured data extraction form using 5 sample studies to ensure clarity and consistency. Six independent reviewers (SR, ZZ, YC, AKP, ML, and SD) then conducted the full data extraction in teams of 2, with each member independently extracting half of the assigned studies. Ambiguities were resolved within the teams to maintain accuracy and consistency. The final data captured study metadata (eg, year of publication and type), dataset characteristics (eg, source, sample size, and type), NLP approaches (eg, models used and task type), SDOH domains addressed, and performance metrics (eg, precision, *F*_1_-score, and recall). We treated these performance metrics as effect measures for outcomes of interest (ie, model performance). Due to heterogeneity in study design, NLP tasks, datasets, and evaluation frameworks, no single standardized effect size metric was applicable, and reported metrics were extracted and summarized descriptively without statistical transformation. Model performance metrics reported by individual studies were extracted as reported and treated descriptively. We summarized study characteristics in structured tables and used visualizations, including a PRISMA flow diagram, frequency plots, heatmaps, and bubble charts, to display study selection, SDOH domains, and NLP model performance patterns. We assessed risk of bias using a short checklist adapted from the Joanna Briggs Institute [42] and covered 6 items: reporting of population demographics (Q1), clarity of study aim (Q2), relevance to SDOH and NLP (Q3), use of a reference standard (Q4), reporting of evaluation measures (Q5), and acknowledgment of study limits (Q6). Each item was rated as yes, no, or unclear. Studies were classified as low (≥5 yes), moderate (3‐4 yes), or high (≤3 yes) risk of bias.

### Influential Studies

To assess visibility and identify recurring methodological, collaborative, and reporting features, we calculated a normalized citation ratio (NCR) for each study:


$$NCR=\frac{\text{Paper`s citations}}{\text{Average of its publication year`s cohort}}$$

This approach adjusts for citation differences based on publication year.

## Results

### Study Selection and Characteristics

**Figure 1.**
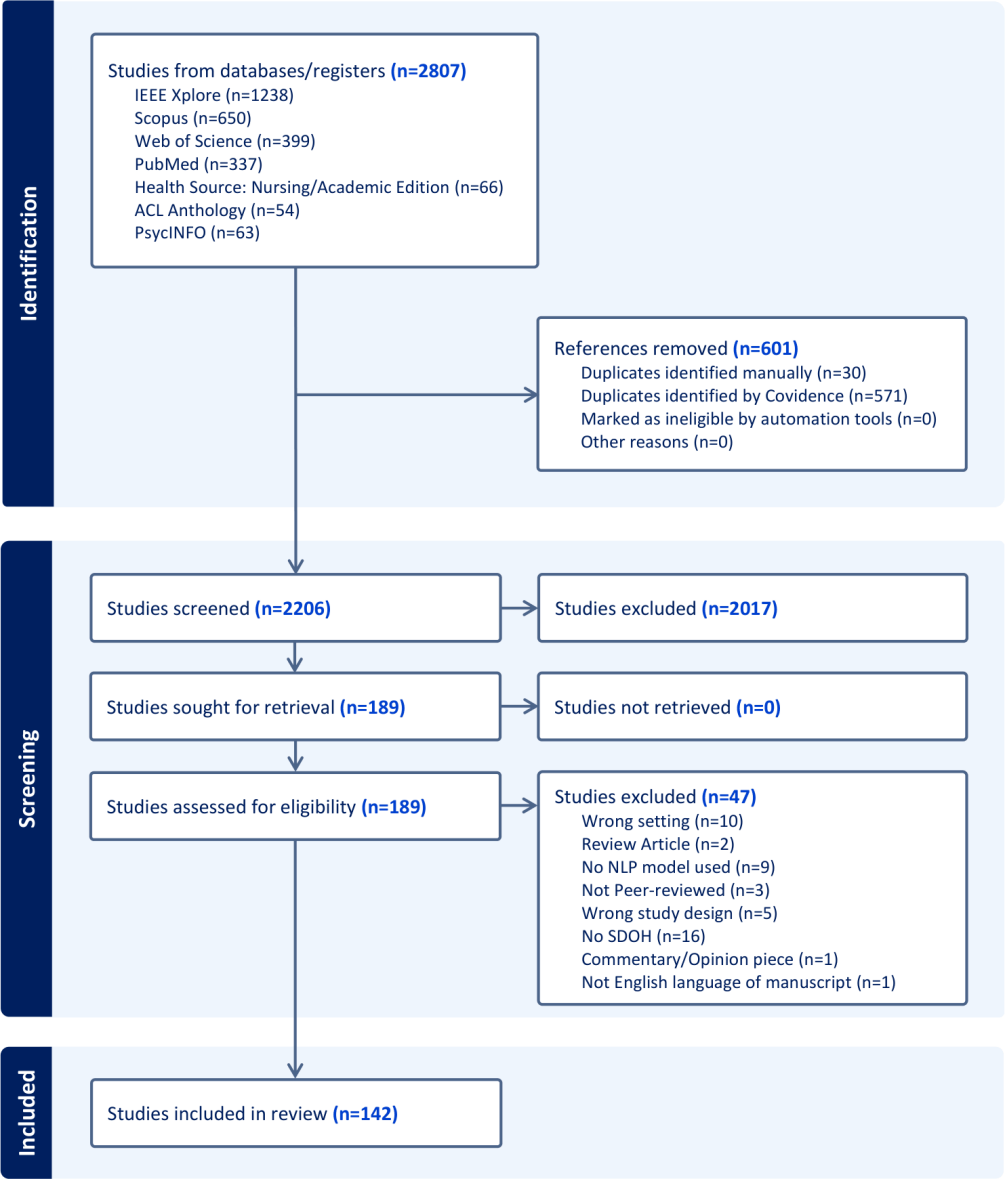
PRISMA (Preferred Reporting Items for Systematic Reviews and Meta-Analyses) flow diagram outlining the inclusion and screening processes for study selection. ACL: Association for Computational Linguistics; NLP: natural language processing; SDOH: social determinants of health.

**Figure 2.**
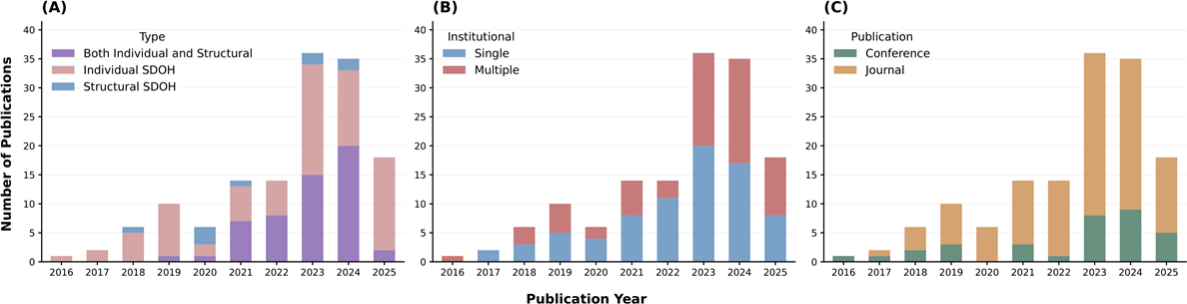
Yearly publication trends in natural language processing for SDOH research illustrate increasing interest over time. (A) SDOH type distribution. (B) Single- versus multi-institution dataset usage. (C) Publication venue distribution. Multi-institution datasets and journal publications increased substantially after 2022. Note that only partial data was available for 2025 at the time of review. SDOH: social determinants of health.

Figure 1 shows the number of articles included in each phase of the screening process, resulting in the eventual inclusion of 142 studies (Multimedia Appendix 1 shows the search strategy results across databases). Figure 2 presents publication trends over the years, illustrating a growing research interest over time, with the majority of studies being published between 2023 and 2025 (89/142, 62.7%). The majority of the studies were published in journals (109/142, 76.7%), while the remaining were in conference or workshop proceedings (Figure 2C). Also, as shown in Figure 2B, single-site studies were more common overall, but there has been a recent rise in multi-site studies (data from multiple institutes). Most studies were rated as low risk of bias (116/142), 26 as moderate, and none as high risk (Table 1). Table S5 in Multimedia Appendix 1 presents a summary of studies. Also, because this review used descriptive narrative synthesis across highly heterogeneous studies, formal heterogeneity analyses, sensitivity analyses, reporting-bias assessment, and certainty-of-evidence grading were not conducted.

**Table 1.** Risk of bias assessment for included studies.

Reference	Q1	Q2	Q3	Q4	Q5	Q6	Total “Y”	Risk category
[43]	✓^a^	✓	✓	✓	✓	✓	6	*↓^b^*
[44]	✓	✓	✓	✓	*•^c^*	✓	5	*↓*
[45]	✓	✓	✓	✓	*•*	✓	5	*↓*
[46]	✓	✓	✓	✓	*•*	✓	5	*↓*
[47]	✓	✓	✓	✓	*•*	✓	5	*↓*
[48]	✓	✓	✓	*×^d^*	*×*	✓	4	*≈^e^*
[49]	✓	✓	✓	✓	*•*	✓	5	*↓*
[50]	*×*	✓	✓	✓	*•*	✓	4	*≈*
[51]	✓	✓	✓	*×*	*•*	✓	4	*≈*
[26]	*×*	✓	✓	✓	✓	✓	5	*↓*
[28]	✓	✓	✓	*×*	*×*	✓	4	*≈*
[52]	✓	✓	✓	✓	✓	✓	6	*↓*
[53]	*×*	✓	✓	✓	✓	✓	5	*↓*
[54]	*×*	✓	✓	*×*	*•*	✓	3	*≈*
[55]	*×*	✓	✓	✓	✓	✓	5	*↓*
[56]	✓	✓	✓	✓	✓	✓	6	*↓*
[57]	✓	✓	✓	✓	✓	✓	6	*↓*
[30]	✓	✓	✓	✓	✓	✓	6	*↓*
[58]	*×*	✓	✓	✓	✓	✓	5	*↓*
[59]	*×*	✓	✓	✓	*•*	✓	4	*≈*
[22]	*×*	✓	✓	✓	✓	✓	5	*↓*
[60]	✓	✓	✓	✓	*•*	✓	5	*↓*
[61]	✓	✓	✓	✓	✓	✓	6	*↓*
[62]	✓	✓	✓	✓	✓	✓	6	*↓*
[63]	✓	✓	✓	✓	*•*	✓	5	*↓*
[23]	✓	✓	✓	✓	✓	✓	6	*↓*
[64]	✓	✓	✓	✓	*•*	✓	5	*↓*
[65]	✓	✓	✓	✓	*•*	✓	5	*↓*
[66]	✓	✓	✓	✓	*•*	✓	5	*↓*
[67]	✓	✓	✓	✓	*•*	✓	5	*↓*
[29]	✓	✓	✓	✓	*•*	✓	5	*↓*
[68]	✓	✓	✓	✓	✓	✓	6	*↓*
[69]	✓	✓	✓	✓	✓	✓	6	*↓*
[70]	*×*	✓	✓	✓	*•*	✓	4	*≈*
[31]	*×*	✓	✓	✓	*•*	✓	4	*≈*
[71]	✓	✓	✓	✓	✓	✓	6	*↓*
[72]	✓	✓	✓	✓	*•*	✓	5	*↓*
[73]	✓	✓	✓	✓	*•*	✓	5	*↓*
[74]	*×*	✓	✓	✓	✓	✓	5	*↓*
[75]	✓	✓	✓	✓	*×*	✓	5	*↓*
[76]	*×*	✓	✓	✓	✓	✓	5	*↓*
[77]	*×*	✓	✓	✓	✓	✓	5	*↓*
[78]	*×*	✓	✓	✓	✓	✓	5	*↓*
[79]	✓	✓	✓	✓	*•*	✓	5	*↓*
[80]	*×*	✓	✓	✓	✓	✓	5	*↓*
[81]	*×*	✓	✓	✓	✓	✓	5	*↓*
[82]	✓	✓	✓	✓	✓	✓	6	*↓*
[83]	✓	✓	✓	✓	*•*	✓	5	*↓*
[32]	*×*	✓	✓	✓	✓	✓	5	*↓*
[84]	*×*	✓	✓	✓	✓	✓	5	*↓*
[85]	*×*	✓	✓	✓	✓	✓	5	*↓*
[86]	*×*	✓	✓	✓	✓	✓	5	*↓*
[87]	*×*	✓	✓	✓	✓	✓	5	*↓*
[88]	✓	✓	✓	*×*	*•*	✓	4	*≈*
[27]	*×*	✓	✓	✓	✓	✓	5	*↓*
[89]	✓	✓	✓	✓	*•*	✓	5	*↓*
[25]	✓	✓	✓	✓	*•*	✓	5	*↓*
[90]	*×*	✓	✓	✓	✓	✓	5	*↓*
[91]	✓	✓	✓	✓	*•*	✓	5	*↓*
[92]	*×*	✓	✓	✓	✓	✓	5	*↓*
[93]	✓	✓	✓	✓	✓	✓	6	*↓*
[94]	✓	✓	✓	✓	✓	✓	6	*↓*
[95]	✓	✓	✓	✓	*×*	✓	5	*↓*
[96]	✓	✓	✓	✓	*×*	✓	5	*↓*
[97]	✓	✓	✓	✓	*•*	✓	5	*↓*
[98]	✓	✓	✓	✓	✓	✓	6	*↓*
[99]	*×*	✓	✓	✓	✓	✓	5	*↓*
[100]	*×*	✓	✓	✓	✓	✓	5	*↓*
[101]	*×*	✓	✓	✓	✓	✓	5	*↓*
[102]	✓	✓	✓	✓	✓	✓	6	*↓*
[103]	✓	✓	✓	*×*	*×*	✓	4	*≈*
[104]	*×*	✓	✓	✓	*•*	✓	4	*≈*
[20]	✓	✓	✓	✓	*•*	✓	5	*↓*
[105]	✓	✓	✓	✓	*×*	✓	5	*↓*
[106]	✓	✓	✓	✓	✓	✓	6	*↓*
[21]	✓	✓	✓	✓	*•*	✓	5	*↓*
[107]	✓	✓	✓	✓	*•*	✓	5	*↓*
[108]	✓	✓	✓	✓	✓	✓	6	*↓*
[109]	*×*	✓	✓	✓	*×*	✓	4	*≈*
[110]	*×*	✓	✓	✓	*•*	✓	4	*≈*
[111]	*×*	✓	✓	✓	✓	✓	5	*↓*
[112]	*×*	✓	✓	*×*	*×*	✓	3	*≈*
[113]	*×*	✓	✓		*×*	✓	3	*≈*
[114]	✓	✓	✓	✓	✓	✓	6	*↓*
[115]	*×*	✓	✓	✓	*•*	✓	4	*≈*
[116]	*×*	✓	✓	✓	✓	✓	5	*↓*
[117]	✓	✓	✓	✓	*•*	✓	5	*↓*
[118]	*×*	✓	✓	✓	✓	✓	5	*↓*
[12]	*×*	✓	✓	✓	✓	✓	5	*↓*
[119]	✓	✓	✓	✓	✓	✓	6	*↓*
[120]	*×*	✓	✓	✓	*•*	✓	4	*≈*
[121]	✓	✓	✓	✓	*•*	✓	5	*↓*
[122]	*×*	✓	✓	✓	✓	✓	5	*↓*
[123]	*×*	✓	✓	✓	✓	✓	5	*↓*
[124]	*×*	✓	✓	✓	*×*	✓	4	*≈*
[125]	*×*	✓	✓	✓	✓	✓	5	*↓*
[126]	*×*	✓	✓	✓	✓	✓	5	*↓*
[127]	*×*	✓	✓	✓	*•*	✓	4	*≈*
[128]	*×*	✓	✓	✓	✓	✓	5	*↓*
[129]	*×*	✓	✓	*×*	*•*	✓	3	*≈*
[130]	✓	✓	✓	✓	✓	✓	6	*↓*
[131]	*×*	✓	✓	✓	*•*	✓	4	*≈*
[132]	✓	✓	✓	✓	✓	✓	6	*↓*
[133]	✓	✓	✓	✓	*•*	✓	5	*↓*
[134]	✓	✓	✓	✓	✓	✓	6	*↓*
[135]	*×*	✓	✓	✓	✓	✓	5	*↓*
[136]	✓	✓	✓	✓	*•*	✓	5	*↓*
[137]	*×*	✓	✓	✓	✓	✓	5	*↓*
[138]	*×*	✓	✓	✓	*•*	✓	4	*≈*
[139]	*×*	✓	✓	✓	✓	✓	5	*↓*
[140]	*×*	✓	✓	✓	*•*	✓	4	*≈*
[141]	✓	✓	✓	✓	✓	✓	6	*↓*
[142]	✓	✓	✓	✓	✓	✓	6	*↓*
[143]	*×*	✓	✓	✓	✓	✓	5	*↓*
[144]	*×*	✓	✓	✓	✓	✓	5	*↓*
[145]	*×*	✓	✓	✓	✓	✓	5	*↓*
[146]	✓	✓	✓	✓	*•*	✓	5	*↓*
[147]	*×*	✓	✓	✓	✓	✓	5	*↓*
[148]	✓	✓	✓	✓	✓	✓	6	*↓*
[149]	*×*	✓	✓	✓	✓	✓	5	*↓*
[150]	✓	✓	✓	✓	✓	✓	6	*↓*
[151]	✓	✓	✓	✓	✓	✓	6	*↓*
[152]	*×*	✓	✓	✓	✓	✓	5	*↓*
[153]	✓	✓	✓	✓	✓	✓	6	*↓*
[154]	*×*	✓	✓	✓	*•*	✓	4	*≈*
[155]	*×*	✓	✓	✓	✓	✓	5	*↓*
[156]	*×*	✓	✓	✓	✓	✓	5	*↓*
[157]	✓	✓	✓	✓	✓	✓	6	*↓*
[158]	✓	✓	✓	✓	✓	✓	6	*↓*
[24]	✓	✓	✓	✓	✓	✓	6	*↓*
[159]	*×*	✓	✓	✓	✓	✓	5	*↓*
[160]	*×*	✓	✓	✓	✓	✓	5	*↓*
[161]	*×*	✓	✓	✓	✓	✓	5	*↓*
[162]	*×*	✓	✓	✓	✓	✓	5	*↓*
[163]	✓	✓	✓	✓	✓	✓	6	*↓*
[164]	✓	✓	✓	✓	✓	✓	6	*↓*
[165]	✓	✓	✓	✓	✓	✓	6	*↓*
[166]	✓	✓	✓	✓	*•*	✓	5	*↓*
[167]	✓	✓	✓	✓	✓	✓	6	*↓*
[168]	✓	✓	✓	✓	✓	✓	6	*↓*
[169]	✓	✓	✓	✓	*•*	✓	5	*↓*
[170]	✓	✓	✓	✓	*•*	✓	5	*↓*

a ✓: Yes.

b *↓*: Low risk of bias.

c *•*: Unclear.

d *×*: No.

e *≈*: Moderate risk of bias.

### Data Sources and Characteristics

Across the 142 studies reviewed, over half used private datasets (81/142, 57.04%), while publicly accessible datasets were used in 29 studies (20.42%). A smaller number of studies used datasets that were available via specific data use agreements (7/142, 4.93%), accessible only for shared tasks (5/142, 3.52%), or available in partial form (subset available, 5/142, 3.52%). The most common data type was EHRs, reported in 93 studies. Eight studies used national datasets (Primary Land Use Tax Lot Output [PLUTO], CalEnviroScreen 4.0, etc). Social media sources, including Twitter, Reddit, and Facebook, were leveraged in 9 studies (5.1%). Other, less frequently reported types included research abstracts, web content, and interviews.

The majority of private datasets included institution-specific EHRs (eg, Columbia University Irving Medical Center; University of California, San Francisco; Johns Hopkins; Eskenazi; Medical University of South Carolina; UF Health; UNC; Kaiser Permanente Southern California) and clinical data warehouses tied to academic or health systems. The Veterans Affairs health system was particularly prominent, covering datasets such as: Veterans Affairs Corporate Data Warehouse [46,47,75,83,89,90,94,135], Veteran Health Administration notes [114], Supportive Services for Veteran Families [75], administrative data [136], Veterans Aging Cohort Study [141]. Several datasets incorporated augmented sources to enrich available information, such as LexisNexis, geospatial SDOH (from diverse government sources [47]), and patient-reported tools (eg, Timeline Follow-Back [83]). Publicly available datasets included Medical Information Mart for Intensive Care III (MIMIC-III) [31,32,55,57,58,74,77,78,82,85,86,95,97,104,118,125,128,130], n2c2 2018/2022 [55,58,80,85,128], SemEval-2015 [128], LGBTQ+ Minority Stress on Social Media (MiSSoM+) dataset [92], and others. MIMIC-III and its derivatives (eg, social and behavioral determinants of health-MIMIC) were frequently used for training [85,86,95], validation [82], or annotation purposes [32].

### SDOH Factors

**Figure 3.**
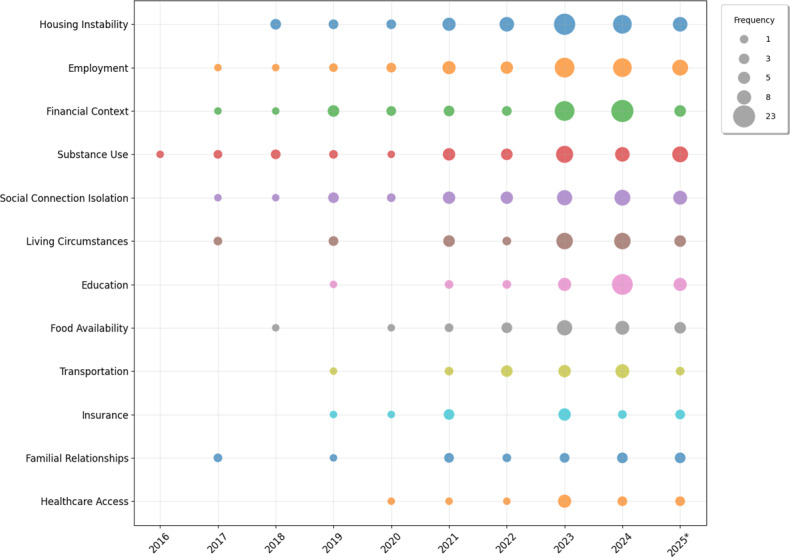
Distribution of the most studied social determinants of health over the years. Bubble size is proportional to the number of studies. Individual-level determinants are frequently studied compared with structural determinants. Table S2 in Multimedia Appendix 1 for the category dictionary. *partial data for 2025.

Figure 3 illustrates the most commonly studied SDOH factors across the years. We show factors that appeared in at least 15 papers. Table 2 shows the SDOH type (individual vs structural) studied in our cohort of papers. The most studied SDOH factors were housing instability (50.7%) and employment (45.8%), appearing in nearly half of the 142 papers, followed by financial context (44.4%), substance use (37.3%), and social isolation (34.5%). Education and living circumstances each appeared in 27.5% and 28.9% of studies, respectively. At the lower end, justice system involvement and language literacy each appeared in approximately 8% of studies. The least studied factors included immigration status (3.5%) and 4 factors that appeared in less than 3 papers each: access to lethal means, acculturation, digital divide, and military sexual trauma. Collectively, these results show that individual SDOH (substance use, social connection/isolation, etc) received more research attention than structural SDOH (transportation, insurance, etc) over the years. The uneven focus on certain SDOH could be due to data and methodological constraints rather than importance alone. Individual-level factors such as housing and employment are more explicitly documented and easier to annotate, whereas structural determinants (eg, immigration status or the digital divide) are often inconsistently recorded or absent from clinical text.

**Table 2.** Social Determinants of Health (SDOH) type distribution.

SDOH type	Reference
Individual (n=79)	[43,111,115-117,119,122-125,127,138,143,144], [12,20,87,93,94,98,99,101,114,118,121,130,136,141,142], [22,23,29,31,45,47,55,57,58,62,70,71,73,74,78,80-83,85,86,90,132-134,137,145-147,149,150,152], [24,135,148,155-166,168-170]
Structural (n=9)	[48,53,59,88,110,112,120,129,126]
Both (n=54)	[21,44,46,95,96,100,102-109,113], [25,27,32,52,56,63,64,67,75-77,79,84,89,91,92,97], [26,28,30,49-51,54,60,61,65,66,68,69,72,128,131,139,140,151,153,154,167]

### NLP Methods and Performance

**Figure 4.**
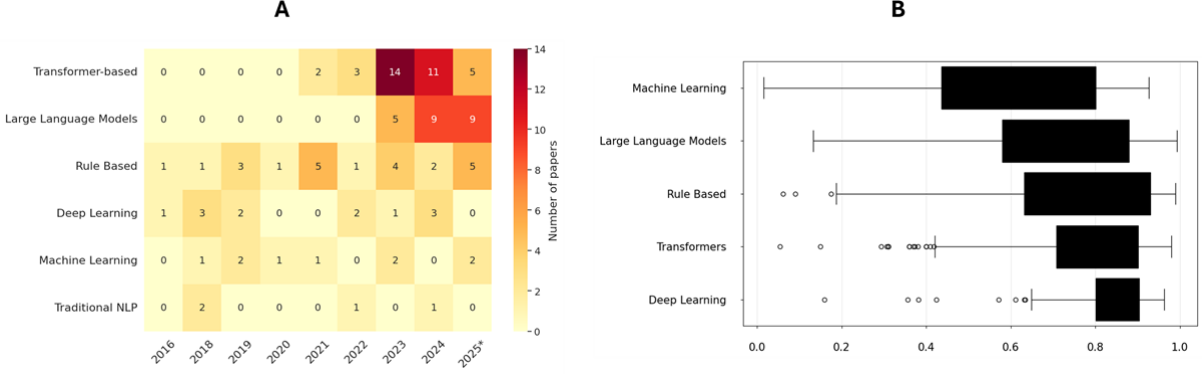
(A) Frequency heatmap representative of the best-performing NLP methodologies across years. Interestingly, transformer- and large language model–based studies surged over the last 3 years. (B) Box plot showing *F*_1_-score distribution by model category. Models with fewer than 20 samples are excluded, and extreme values are removed for readability. Table S3 in Multimedia Appendix 1 details the category dictionary. *partial data for 2025. NLP: natural language processing.

**Figure 5.**
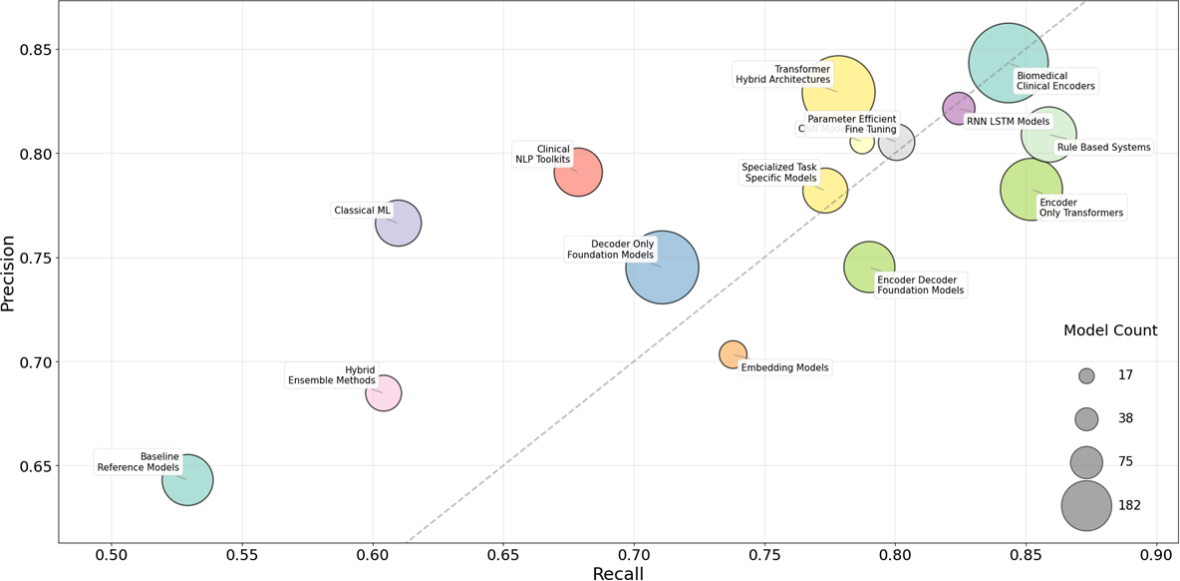
Bubble chart representing mean values of precision and recall for different NLP model categories. The dashed line represents the equal-precision and recall boundary. Bubble size is proportional to the number of models; only categories with ≥ 7 observations are shown. Performance metrics are from heterogeneous evaluation contexts, representative of aggregate trends, and are not individually comparable across studies. Table S4 in Multimedia Appendix 1 details NLP method categorization. LSTM: long short-term memory; ML: machine learning; NLP: natural language processing; RNN: recurrent neural network.

Figure 4A presents a heatmap illustrating the temporal distribution and frequency of the dominant NLP methods identified in this review. Among the included studies, the majority (n=83) quantified model performance using standard metrics, including *F*_1_-score, precision, and recall. Figure 4B delineates the distribution of *F*_1_-scores across the 5 categories, highlighting significant performance variations between rule-based, traditional machine learning, and deep learning approaches. Complementing this, Figure 5 provides a bubble chart depicting the mean precision and recall values for each model category. Collectively, the visualized performance trends show the general capabilities of different modeling approaches for SDOH tasks, though performance may vary based on task complexity, dataset characteristics, and annotation quality.

Overall, the results highlight that transformer-based encoder-only models such as BERT and generative models (encoder-decoders) trained on specialized clinical tasks currently represent the state of the art in terms of SDOH classification and extraction performance. LLMs (19.7% of reviewed studies, including encoder-decoders) and their capabilities represent an emerging area of research. The limited adoption of LLMs likely reflects practical and ethical concerns rather than a lack of capability. Challenges related to hallucinations, limited reproducibility, fairness and bias risks, and uncertainty around data leakage and governance (especially in clinical settings) may constrain their broader use despite strong performance in controlled evaluations. The majority of studies included in this review (≈90%) use a cross-sectional design, focusing on extracting or classifying SDOH information from a single point in time, providing a valuable snapshot. While useful, such approaches cannot capture the changes in SDOH factors among study subjects. Only a handful of studies [66,75,83] adopt a longitudinal approach, following individuals’ records (like EHRs) over time to track changes in SDOH and their impact on health outcomes.

### Model Interpretation Techniques

Model interpretation is essential for understanding not just how well a model performs, but also the basis for its decisions. A total of 82 studies reported on using interpretability techniques to gain insights into model performance. These included qualitative content analysis [29,59,131], Shapley additive explanations [47,71,163], Local Interpretable Model-Agnostic Explanations [23], attention visualization or neuron activation analysis [54], and ablation studies [57,87]. One common approach found across studies was error analysis, which plays a key role in understanding how and why models make specific mistakes. It involves examining misclassifications or incorrect outputs to identify patterns of failure. Several studies in our review performed manual (domain expert-based) error analysis to identify model weaknesses. For instance, one paper found that their model struggled to distinguish between general and specific substance-related terms by incorrectly extracting the generic verb “smokes” as the key information when the actual target was the more specific noun “cigarettes” [77]. Another study showed that soft prompting (using trainable continuous vectors instead of manually crafted text instructions) improved the extraction of overlapping or nested SDOH concepts, especially when prompt length was tuned [58]. Error analyses were particularly prominent in studies using LLMs such as ChatGPT, Llama, and Gemini [26,31,57,59,61,155-157,159] because these models (being black-box) can produce outputs that appear coherent and convincing even when they contain mistakes. In addition, confusion matrices were used to visualize class-level misclassification patterns and highlight systematic errors [62].

### Influential Studies

Analysis of the 20 influential studies published between 2018 and 2025 reveals a methodological landscape dominated by advanced NLP architectures, including BERT [60,78,80,118,130] and LLMs [57], alongside some rule-based [22,127] and hybrid approaches [27,109,168]. Notably, three-quarters (15/20) of these studies relied on manual annotation or clinician input to establish ground truth, highlighting a continued reliance on human expertise to ensure data quality and study robustness. The prevalence of multi-institutional collaborations in these studies (n=11) suggests that diverse teams and broader data sources are key drivers of research impact, likely facilitating increased generalizability and resource access. Regarding performance, the majority of studies (13/20) reported strong *F*_1_-scores, though results varied by task complexity. High benchmarks included concept extraction scores up to 0.9118, and relation extraction at 0.8332 [58], with Seq2Seq models achieving approximately 0.889 [80] and other systems reaching 0.86 [78]. Performance heterogeneity was evident in class-specific tasks; for example, one study reported attribute performance (eg, substance use and employment) ranging from 0.81 to 0.93 [118], while another reported a wider spread from 0.491 for non-SDOH factors to 0.774 for the best-performing class (occupation) using the same model [130]. Beyond standard metrics, one study involving EHR data reported that NLP-based methodology captured SDOH in 80.03% of cases compared with 38.17% using structured fields only [89]. Surprisingly, only 9 out of these 20 studies provided publicly accessible code. Although this represents a higher proportion than the overall set (49/142, 34.5% papers shared their code), public code availability remains limited even among the most influential studies.

### Publishing Venues and Funding Sources

The most frequent publication venue was the Journal of the American Medical Informatics Association (12/142, 8.5%), followed by Journal of the American Medical Informatics Association Open (4.9%), Journal of Medical Internet Research (4.2%), and Journal of Biomedical Informatics (4.2%). Conference contributions were most often from the IEEE and AMIA Annual Symposium Proceedings. A majority of studies (110/142, 77.46%) acknowledged funding. The National Institutes of Health and its affiliated institutes (National Library of Medicine, National Center for Advancing Translational Sciences, National Cancer Institute, National Institute on Aging, and NIDA) were the leading funders (64/110, 58.2% of funded studies). Other key funders included the National Science Foundation (n=10), Agency for Healthcare Research and Quality (n=6), and international agencies such as the National Institute for Health Research and Canadian Institutes of Health Research. There is an increase in the number of studies reporting federal funding for SDOH-related NLP research in recent years. The percentage of funded studies more than doubled from 33% to 67% during 2018‐2020, to 78%‐83% during 2021‐2025.

## Discussion

### Principal Findings

This systematic review synthesizes evidence from 142 studies, addressing 2 primary objectives: to characterize NLP techniques, including LLMs, used to analyze SDOH in text-based data, and to assess their reported effectiveness, gaps, and future research needs. Overall, the review reveals rapid methodological advancement and improvement in NLP performance in recent years, marked by a shift toward transformer-based architectures and emerging use of LLMs, with most applications focused on extracting or classifying individual-level SDOH from clinical and social text. Despite technical progress, the review shows that challenges related to data availability, performance reproducibility, model interpretability, and translation into real-world clinical or public health practice remain. Key findings are elaborated in the following paragraphs.

Across studies, model performance in identifying individual SDOH from unstructured text has generally improved over time, reflecting advances in representation learning and modern NLP systems’ ability to capture abstract and context-dependent social concepts [171]. These advances are likely driven by both increased recognition from the research community of the role of SDOH in shaping health outcomes [172] and by the maturation of transformer-based models that perform well on complex linguistic tasks [173]. Strong reported performance, however, does not consistently translate into reproducible findings or application-ready systems [174]. Many studies relied on private, institution-specific EHR datasets, which limit independent replication and cross-site validation [175]. Although manual and clinician-led annotation was common across the reviewed studies, detailed documentation of annotation guidelines and procedures was often lacking, making it difficult to assess consistency in how SDOH concepts were defined and applied across studies. Limited transparency around data and annotation practices also has important equity implications [176]. Reliance on proprietary EHR data favors well-resourced institutions and restricts broader participation in SDOH-focused NLP research [177].

This review also sheds light on the gap between technical development and practical implementation, and limitations on model interpretability. While many studies report promising model performance, few detail pathways for integrating NLP-derived SDOH insights into clinical workflows or public health interventions, which may be due to systemic, technical, and regulatory barriers [178]. As a result, the potential of these methods to inform equity-focused decision-making remains largely unrealized, a finding supported by prior research [176]. NLP/LLM model interpretability has received more research attention in recent years [179]. A little over half of all reviewed studies used some variant of interpretability or explainability methods, but their use was inconsistent and often inadequately discussed. This gap is relatively more critical for SDOH applications, where language may reflect complex social, cultural, and structural nuances, with uninterpretable errors disproportionately affecting marginalized groups [180]. Limited interpretability also undermines trust in systems’ ability to inform care or policy, both within and outside the sphere of SDOH [181].

### Future Research Directions

Based on the review, we outline several priorities for advancing NLP and LLM applications in the study of SDOH. Most of the existing work used cross-sectional designs, offering only static snapshots of social context. Future research should adopt longitudinal designs capable of capturing the dynamic nature of social risk factors, their accumulation over time, and their interaction with health trajectories [182]. Such longitudinal approaches can include continuous monitoring as well as repeated snapshot or point-in-time measures collected at predetermined intervals [183], for tracking meaningful changes in SDOH. Future work may combine unstructured narratives with structured EHR variables, imaging, and community-level data to produce more holistic and actionable models [184,185]. Effective multimodal approaches must account for heterogeneity in SDOH-containing data [186] to maximize information capture and analytical utility. Advancing the field will also require stronger commitments to transparency and reproducibility. Establishing open benchmarks, shared tasks, and community challenges has driven NLP method development on many topics [80,128], and should be the focus of future work to promote transparency and reproducibility in SDOH-related NLP. Greater attention is also needed for structural determinants of health. Fewer than one-fifth of reviewed studies focused on structural SDOH factors such as policy, racism, or socioeconomic stratification [187]. Capturing structural determinants presents unique methodological challenges, such as patient-reported data on systemic factors being limited by awareness and subjectivity [188]. Linking individual-level health narratives with community- and policy-level data may offer a more comprehensive approach to understanding how structural forces shape health outcomes in future research [189]. Bias and ethical considerations also remain underexplored. Few studies systematically examined algorithmic bias, despite well-documented risks of inequities in automated decision-making [190,191]. Future systems must embed safeguards for fairness, inclusivity, and accountability to avoid reinforcing disparities. Recent work has proposed actionable frameworks and techniques for mitigating bias in artificial intelligence systems [192-194]. Adopting such approaches will be essential for ensuring SDOH-focused models promote rather than perpetuate health inequities. In addition, while clinical concepts are supported by structured hierarchies such as the Unified Medical Language System [195], no equivalent framework exists for social (or nonclinical) determinants. Establishing such frameworks would reduce inconsistencies, enable interoperability, and support the creation of benchmark datasets. Finally, improving model generalizability remains critical, as models trained on single-institution EHRs often underperform in external settings, reflecting the lexical, demographic, and contextual variability of clinical narratives [174,196]. Future work should therefore move beyond single-site training by explicitly benchmarking models in cross-institution evaluations [197]. Training on pooled multi-site data and testing on held-out institutions, with performance reported by SDOH category and population subgroup, may provide actionable evidence of real-world generalizability.

### Limitations

This review has several limitations. Determining whether studies were directly relevant to SDOH required subjective judgment, as many did not explicitly frame their research in SDOH terms. This introduces potential selection bias, despite a structured review process. To balance comprehensiveness and feasibility, we adhered to predefined selection criteria, focusing primarily on studies with a clear connection to SDOH. Although this approach helped maintain relevance, it may have led to the exclusion of studies with indirect but meaningful contributions to the field. Future work could explore more systematic methods, such as automated screening tools or expert consensus frameworks, to enhance the consistency and reproducibility of the selection process. We also acknowledge that our synthesis is constrained by heterogeneity across study designs, SDOH definitions, annotation schemes, datasets, and evaluation metrics, which limits direct comparability of reported performance across studies.

### Conclusions

In this systematic review, we provide a comprehensive synthesis of NLP and LLM applications for SDOH by systematically surveying methodologies, data sources, evaluation practices, and translational readiness across 142 studies. In contrast to prior reviews that covered subdomains of SDOH, clinical contexts, or individual modeling techniques, ours provides a comparison of tasks, architectures, performance patterns, interpretability practices, and reproducibility considerations across the research spectrum, spanning both individual and structural SDOH. By discussing NLP/LLM technical performance in the broader context of data accessibility, bias, generalizability, and implementation, this review advances the field beyond proof-of-concept model development toward a clearer understanding of what is required for real-world deployment. The findings highlight that, while NLP-based SDOH extraction is technically mature for certain use cases, its impact remains limited by fragmented SDOH representations, a paucity of public benchmarks, and insufficient evaluation in clinical and public health workflows. In light of our findings, future work should prioritize public benchmarks, clearer reporting, and more inclusive datasets to advance this emerging field. Addressing these gaps has direct real-world implications by facilitating inclusive data practices, interpretable and reproducible methods, and implementation-focused evaluations.

## Supplementary material

10.2196/83793 Multimedia Appendix 1 Search queries, results, and categorization; summary of studies.

10.2196/83793 Checklist 1 PRISMA checklist.

## Abbreviations

EHR: electronic health record
LLM: large language model
MIMIC: Medical Information Mart for Intensive Care
MiSSoM+: Minority Stress on Social Media
NCR: normalized citation ratio
NLP: natural language processing
PLUTO: Primary Land Use Tax Lot Output
PRISMA: Preferred Reporting Items for Systematic Reviews and Meta-Analyses
SDOH: social determinants of health
