# Supplementary material for: Applications of Natural Language Processing and Large Language Models for Social Determinants of Health: Systematic Review
Source: J Med Internet Res. 2026 Apr 28;28:e83793. doi: 10.2196/83793 (PMC13123760; doi:10.2196/83793)
Supplement: Multimedia Appendix 1 [file jmir-v28-e83793-s001.pdf]

Supplementary material

A Search Queries

A.1 ACL

The records sent to covidence do not include ACL Anthology as there's no way to batch export records. A team member (SR) searched ACL Anthology and entered this string in the search box:

"Social Determinants of Health" OR SDOH OR SDH OR SBDH* OR "determinants of health" OR "health determina*" OR "life events" OR "social determinant*" OR "socioeconomic determinant*" OR "socioeconomic factor*" OR "social determinate*" OR "social factor*" OR "social need*" OR "social prescribing" OR "social determining factor*" OR "social risk*" and then downloaded results as appropriate using zotero.

A.2 PubMed

("Natural Language Processing"[Mesh] OR "natural language"[tw] OR NLP[tw] OR "large LM*"[tw] OR LLM[tw] OR LLMs[tw] OR "large language model*"[tw] or ChatGPT*[tw] OR "Chat GPT*"[tw] OR GPT4*[tw] OR GPT-4*[tw] OR GPT3*[tw] OR GPR-3*[tw] OR "Generative Pre-trained Transformer*"[tw] OR LLAMA[tw] OR "Claude 3"[tw] OR Mistral[tw] OR MedPaLM*[tw] OR Med-PaLM*[tw] OR "text mining"[tw] OR "text process*"[tw] OR "information retrieval"[tw] OR "information extract*"[tw])

AND

("Social Determinants of Health"[Mesh] OR SDOH[tw] OR SDH[tw] OR SBDH*[tw] OR "determinants of health"[tw] OR "health determina*"[tw] OR "life events"[tw] OR "social determinant*"[tw] OR "socioeconomic determinant*"[tw] OR "socioeconomic factor*"[tw] OR "social determinate*"[tw] OR "social factor*"[tw] OR "social need*"[tw] OR "social prescribing"[tw] OR "social determining factor*"[tw] OR "social risk*"[tw])

Filters: English, 2014-2025, Exclude Preprints

A.3 Web of Science

("natural language" OR NLP OR "large LM*" OR LLM OR LLMs OR "large language model*" OR ChatGPT* OR "Chat GPT*" OR GPT4* OR GPT-4* OR GPT3* OR GPR-3* OR "Generative Pre-trained Transformer*" OR LLAMA OR "Claude 3" OR Mistral OR MedPaLM* OR Med-PaLM* OR "text mining" OR "text process*" OR "information retrieval" OR "information extract*")

AND

("Social Determinants of Health" OR SDOH OR SDH OR SBDH* OR "determinants of health" OR "health determina*" OR "life events" OR "social determinant*" OR "socioeconomic determinant*" OR "socioeconomic factor*" OR "social determinate*" OR "social factor*" OR "social need*" OR "social prescribing" OR "social determining factor*" OR "social risk*")

Filters: English, 2014 to 2025, Document Types - Article, Proceeding Paper, Early Access, Data Paper

A.4 IEEE Xplore

("natural language" OR NLP OR "large LM" OR LLM OR LLMs OR "large language model" OR "large language models" OR ChatGPT OR "Chat GPT" OR GPT4 OR GPT-4 OR GPT3 OR GPR-3 OR "Generative Pre-trained Transformer" OR LLAMA OR "Claude 3" OR Mistral OR MedPaLM OR Med-PaLM OR "text mining" OR "text process*" OR "information retrieval" OR "information extraction") AND ("Social Determinants of Health" OR SDOH OR SDH OR SBDH OR "determinants of health" OR "health determinants" OR "life events" OR "social determinants" OR "socioeconomic determinants" OR "socioeconomic factor*" OR "social determinates" OR "social factor*" OR "social need*" OR "social prescribing" OR "social determining factor*" OR "social risk" OR "social risks")

Filters: 2014 to 2025, Journals

A.5 Scopus

(“natural language” OR NLP OR “large LM*” OR LLM OR LLMs OR “large language model*” OR ChatGPT* OR “Chat GPT*” OR GPT4* OR GPT-4* OR GPT3* OR GPR-3* OR “Generative Pre-trained Transformer*” OR LLAMA OR “Claude 3” OR Mistral OR MedPaLM* OR Med-PaLM* OR “text mining” OR “text process*” OR “information retrieval” OR “information extract*”)

AND

("Social Determinants of Health" OR SDOH OR SDH OR SBDH* OR “determinants of health” OR “health determina*” OR “life events” OR “social determinant*” OR “socioeconomic determinant*” OR “socioeconomic factor*” OR “social determinate*” OR “social factor*” OR “social need*” OR “social prescribing” OR “social determining factor*” OR “social risk*”)

Filters: 2014-2025, English, Article and Conference Paper

A.6 PyscINFO

(“natural language” OR NLP OR “large LM*” OR LLM OR LLMs OR “large language model*” OR ChatGPT* OR “Chat GPT*” OR GPT4* OR GPT-4* OR GPT3* OR GPR-3* OR “Generative Pre-trained Transformer*” OR LLAMA OR “Claude 3” OR Mistral OR MedPaLM* OR Med-PaLM* OR “text mining” OR “text process*” OR “information retrieval” OR “information extract*”) AND ("Social Determinants of Health" OR SDOH OR SDH OR SBDH* OR “determinants of health” OR “health determina*” OR “life events” OR “social determinant*” OR “socioeconomic determinant*” OR “socioeconomic factor*” OR “social determinate*” OR “social factor*” OR “social need*” OR “social prescribing” OR “social determining factor*” OR “social risk*”) Filters: 2014-2025, English, Academic Journals and Dissertations

A.7 Health Source: Nursing/Academic Edition

(“natural language” OR NLP OR “large LM*” OR LLM OR LLMs OR “large language model*” OR ChatGPT* OR “Chat GPT*” OR GPT4* OR GPT-4* OR GPT3* OR GPR-3* OR “Generative Pre-trained Transformer*” OR LLAMA OR “Claude 3” OR Mistral OR MedPaLM* OR Med-PaLM* OR “text mining” OR “text process*” OR “information retrieval” OR “information extract*”) AND ("Social Determinants of Health" OR SDOH OR SDH OR SBDH* OR “determinants of health” OR “health determina*” OR “life events” OR “social determinant*” OR “socioeconomic determinant*” OR “socioeconomic factor*” OR “social determinate*” OR “social factor*” OR “social need*” OR “social prescribing” OR “social determining factor*” OR “social risk*”) Filters: 2014-2025, English, Peer-reviewed scholarly journals

B Search Query Results

Table S1 shows the search query results across databases.

Table S1: Search Strategy Results

S.No.	Database Name	Search Date	Number of Records
1	PubMed	11/02/2025	337
2	Web of Science	11/02/2025	399
3	IEEE Xplore	11/02/2025	1238
4	SCOPUS	11/02/2025	650
5	PsycINFO	11/02/2025	63
6	HealthSource: Academic Nursing	11/02/2025	66
7	ACL Anthology	11/02/2025	54
		**Total (Unique)**	**2807**

C Categorization dictionaries

Throughout this paper, we used umbrella terms to categorize various terms into one category for clarity and consistency. Here, we provide comprehensive dictionaries of these categorizations. Each table (S2, S3, S4) lists the overarching category alongside representative terms grouped under it.

Table S2: Comprehensive SDOH categories and related terms.

Category	Terms
Substance use	alcohol, drugs, smoking, tobacco, addiction, substance abuse, opioids, marijuana
Housing instability	homelessness, housing insecurity, eviction, unstable housing, temporary housing
Employment	unemployment, job loss, work status, occupation, employment instability, workplace
Social connection / isolation	loneliness, social support, isolation, community connections, social networks
Education	educational attainment, literacy, school completion, academic achievement
Financial context	income, poverty, economic hardship, financial stress, socioeconomic status
Food availability	food insecurity, hunger, nutrition access, food deserts, food assistance
Living circumstances	household composition, family structure, living conditions, domestic violence
Transportation	access to transportation, mobility, travel barriers, public transit
Insurance	health insurance status, coverage, uninsured, insurance type, Medicaid
Healthcare access	access to medical care, healthcare barriers, medical services availability, provider access
Discrimination	racial discrimination, bias, prejudice, unfair treatment, systemic racism
Environmental factors	pollution, environmental hazards, air quality, toxic exposure, climate, neighborhood conditions
Familial relationships	family dynamics, family support, family conflict, parental relationships, family structure
Justice system involvement	incarceration, criminal justice, legal system, arrest, prison, probation
Language literacy	language barriers, English proficiency, communication barriers, linguistic competency
Immigration status	immigrant status, documentation, visa status, refugee status, citizenship
Access to lethal means	firearm access, weapon availability, means restriction, suicide methods
Acculturation	cultural adaptation, cultural integration, cultural identity, assimilation, cultural stress
Digital divide	internet access, technology access, digital literacy, online resources, digital barriers
Military sexual trauma	MST, military sexual assault, military harassment, veteran trauma, military-related sexual violence

Table S3: Broad NLP methods categorization.

Category	Methods
Deep Learning	BiLSTM, BiLSTM-CNN-CRF (Proposed), BiLSTM-CRF, BiLSTM-CRF-MTL, BLSTMs, BLSTMs+GloVe, BLSTMs+Word2Vec, BNP (BiLSTM-CNN-CRF), CAML, CNN, CNN NER, CNN with word2vec, CNN+LSTM with word2vec, CNNs, CNNs+GloVe, CNNs+Word2Vec, Deep learning algorithm, GRUs, GRUs+GloVe, GRUs+Word2Vec, JSL NER (ner_sdoh_en), LSFA, LSTM, LSTM with word2vec, LSTM_general, LSTM_mimic, LSTMs, LSTMs+GloVe, LSTMs+Word2Vec, RETAIN, RNN, RNNs, RNNs+GloVe, RNNs+Word2Vec, SWCNN with 100-dimensional word2vec, SWCNN with 200-dimensional word2vec, Shallow Neural Network, TLSTM, spaCy's CNN-based NER, word2vec, word2vec (context size = 15), word2vec (context size = 5)
LLM (Decoder-only and Encoder-Decoder)	0-Shot SDoH-GPT, 2-Shot E SDoH-GPT, 2-Shot H+Expl SDoH-GPT, DeepSeek R1, FLAN-T5-XL, Fine-tuned Flan-T5 XL, Fine-tuned Flan-T5 XXL, Flan-T5, Flan-T5 (fine-tuned), Flan-T5 Large, Flan-T5 XL, Flan-T5 XL (gold data only), Flan-T5 XL (with synthetic data), Flan-T5 XL + Semantic Type, Flan-T5 XXL (with synthetic data), Flan-T5 XXL 0-shot, Flan-T5 XXL 5-shot, Flan-T5 XXL finetuned on ANLI, Flan-T5 XXL finetuned on ANLI + SDOH-NLI, Flan-T5 XXL finetuned on SDOH-NLI, Flan-UL2 0-shot, Flan-UL2 5-shot, GPT 3.5, GPT-3.5, GPT-3.5-turbo End2End, GPT-4, GPT-4 (GPT-inline), GPT-4 (GPT-standoff), GPT-4 + 3-shot, GPT-4o, GPT3.5 (zero-shot), GPT4 (10-shot), GTR XXL, GatorTronGPT-20B, GatorTronGPT-20B-Ptuning, GatorTronGPT-5B, GatorTronGPT-5B-Ptuning, Google/flan-ul2 (2-shot), Google/flan-ul2 (zero-shot), LLaMA 2 7B, Llama 3.1 8B, Llama-2-13b, Llama-2-7b, Meta-llama/llama-2-7b-chat (few-shot), Meta-llama/llama-2-7b-chat (zero-shot), Microsoft (T5), Multi-stage LLM Framework, PaLM2, SENTLI, SENTLI, threshold tuned, Sentence-T5 11B, Spark NLP, T5 v1.1 large (SHACM+SHACW), T5 v1.1 large (SHACM→SHACW), T5 v1.1 large (seq2seq), T5-2sQA, T5-Event, T5-large seq2seq, WizardLM-13b, openchat_3.5, vicuna-13b, vicuna-33b, vicuna-7b, zephyr-7b
Rule-based / Dictionary-based	Baseline (Domain Expert), BioMedICUS, EasyCIE Rules-based NLP Engine, EntityRuler module of spaCy 2.3 Python toolkit, FIIESCT (Food Insecurity in Electronic and Social Work Texts), ICD Diagnosis Codes, ICD-10 Code, Keyword Processor, Keyword Search, Linguamatics I2E version 5.3, Manual Chart Review, MetaMap, Moonstone, Moonstone (Rule-based NLP system), NLP application (rule-based), NLP4Eye (Rule-based), NegEx, OHNLP Toolkit with MedTagger, Pattern Matching Baseline, ReHouSED (NLP-derived measure), Refined spaCy PhraseMatcher NLP Algorithm, RegEx, RegEx (Regular Expression), Rule based, Rule-based NLP System, Rule-based NLP algorithm, Rule-based Pipeline, Rule-based System, Rule-based approaches only, UMLS, cTAKES, cTAKES (Default), cTAKES (InfoCommons), cTAKES (SDoH)

Continued on next page

Table S3 – continued from previous page

Category	Methods
Traditional ML	Baseline Model (Structured EHR Only), Conditional random field classifier, DT, DT+TF-IDF, Hybrid (Pattern Matching + Logistic Regression), Hybrid NLP + ML (XGBoost), LR, LR+TF-IDF, Linear SVM, Linear SVM with Bag of Words, Logistic Regression, Logistic Regression (LASSO), Logistic Regression with Keywords, NB+TF-IDF, Note-based (NB) Model, RF, RF+TF-IDF, Random Forest, Random Forest using n-gram, Regularized logistic regression, SVM, SVM+TF-IDF, Structured EHR + Text, Structured EHR Only, Substance Use - Text Only, Support-vector machine-based classifier, Terminology-based (TB) Model, Text Only, Word2Vec with Decision Tree, Word2Vec with Random Forest, Word2Vec with SVC, XGBoost, XGBoost trained on 0-Shot SDoH-GPT annotations, XGBoost trained on 2-Shot E SDoH-GPT annotations, XGBoost trained on 2-Shot H SDoH-GPT annotations, XGBoost trained on 2-Shot H+Expl SDoH-GPT annotations, Yale cTAKES extension (YTEX)
Traditional NLP	Bag of Words (BOW), Baseline + NLP Keyword Model, Baseline + NLP Topic Model (LDA), LSI, Lexical association (Chi-square test), TF-IDF weighted cosine similarity retrieval, WNLing (WordNet + Linguistic), Word2vec-based Information Retrieval with TF-IDF
Transformer-based	ALBERT, BERT, BERT (Original dataset), BERT (no prompt, unfrozen), BERT with Back Translation augmentation, BERT with EDA augmentation, BERT with gpt-3.5-turbo augmentation, BERT with gpt-3.5-turbo-0301 augmentation, BERT with segment encoding, BERT with text-curie-001 augmentation, BERT with text-davinci-003 augmentation, BERT-Base, BERT-BiGRU, BERT-CNN, BERT-MIMIC-MRC, BERT-MRC, BERT-base (gold data only), BERT-based pipeline, BERT-large, BERT__general, BERT__mimic, BioBERT, BioBERT + RoBERTa Joint Trigger and Argument, BioBERT v1.1, BioBERT v1.2, BioBERT-Base v1.0, BioBERT-Base v1.1, BioBERT-Base v1.2, BioBERT-KIRESH, BioBERT-KIRESH-Prompt, BioBERT-Prompt, BioClinical-BERT, BioClinicalBERT, Bio__ClinicalBERT, Bio__ClinicalBERT-KIRESH, Bio__ClinicalBERT-KIRESH-Prompt, Bio__ClinicalBERT-Prompt, BlueBERT, CHOP (BERT), CT-BERT, ClinicalBERT, ClinicalBERT with Decision Tree, ClinicalBERT with Random Forest, DeBERTa, DistilBERT, ELECTRA, Event Extractor (Bio+Discharge Summary BERT + bi-LSTM + CRF), Flair + RoBERTa Joint Trigger and Argument, GatorTron, GatorTron (BERT variant), GatorTron-3.9B (soft prompt, frozen), GatorTron-3.9B (soft prompt, unfrozen), GatorTron-3.9B-Ptuning, GatorTron-345M, GatorTron-345M (soft prompt, unfrozen), GatorTron-345M-Ptuning, GatorTron-8.9B (soft prompt, frozen), GatorTron-8.9B (soft prompt, unfrozen), GatorTron-8.9B-Ptuning, GatorTron-MRC, Hierarchical Longformer, Hierarchical Roberta, IBM (BERT), KEPT, Longformer, MLTB (Multilabel Multitask BERT), Proposed Approach (Context-Specific Feature Augmentation), PubMedBERT, RoBERTa, RoBERTa Classification, RoBERTa Classification (SHACM), RoBERTa Classification (SHACM+IN-HOUSE), RoBERTa Classification (SHACM+SHACW), RoBERTa Entailment, RoBERTa NER, RoBERTa-Ensemble Classification, RoBERTa-MIMIC-MRC, RoBERTa-MIMIC-Trial, RoBERTa-based system, RoBERTa-large, RoBERTa__general, RoBERTa__mimic, SciBERT, Sentence Transformer all-mpnet-base-v2, Sentence-BERT, Surrogate Classifier (Bio+Discharge Summary BERT + bi-LSTM), T5-3B + RoBERTa Joint Trigger and Argument, T5-3B + RoBERTa Overlap Trigger + Overlap Argument, Transformer-based NLP model, Transformer-based NLP model (BERT/GPT), Triaffine Independent Trigger + Argument, Triaffine Joint Trigger + Argument, mSpERT, mSpERT (Experiment 1), mSpERT (Experiment 2), mSpERT (Experiment 3), mSpERT (fine-tuned)

Table S4: Comprehensive NLP models sub-categorization.

Category	Methods
Encoder Only Transformers	BERT, BERT-Base, BERT-large, BERT__general, RoBERTa, RoBERTa-large, RoBERTa__general, ALBERT, ELECTRA, DistilBERT, DeBERTa, Longformer, BERT-based pipeline, BERT with segment encoding, RoBERTa-based system, RoBERTa Classification, RoBERTa Classification (SHACM+SHACW), RoBERTa Classification (SHACM), RoBERTa Classification (SHACM+IN-HOUSE), RoBERTa Entailment, RoBERTa-Ensemble Classification, Hierarchical Roberta, Hierarchical Longformer, BERT (Original dataset), RoBERTa NER, BERT with gpt-3.5-turbo augmentation, BERT with gpt-3.5-turbo-0301 augmentation, BERT with text-davinci-003 augmentation, BERT with Back Translation augmentation, BERT with text-curie-001 augmentation, BERT with EDA augmentation, CHOP (BERT), IBM (BERT)
Biomedical Clinical Encoders	BioBERT, BioBERT-Base v1.0, BioBERT-Base v1.1, BioBERT-Base v1.2, BioBERT v1.1, BioBERT v1.2, BioBERT-KIRESH, BioBERT-Prompt, BioBERT-KIRESH-Prompt, SciBERT, PubMedBERT, BlueBERT, BioClinicalBERT, ClinicalBERT, Bio__ClinicalBERT, BioClinical-BERT, Bio__ClinicalBERT-KIRESH, Bio__ClinicalBERT-Prompt, Bio__ClinicalBERT-KIRESH-Prompt, CT-BERT, BERT__mimic, RoBERTa__mimic, GatorTron, GatorTron-345M, GatorTron (BERT variant)
Decoder Only Foundation Models	GPT-4, GPT-4o, GPT-3.5, GPT 3.5, GPT-3.5-turbo End2End, GPT4 (10-shot), GPT3.5 (zero-shot), GPT-4 + 3-shot, GPT-4 (GPT-inline), GPT-4 (GPT-standoff), PaLM2, Meta-llama/llama-2-7b-chat (zero-shot), Meta-llama/llama-2-7b-chat (few-shot), LLaMA 2 7B, Llama-2-7b, Llama-2-13b, Llama 3.1 8B, GatorTronGPT-5B, GatorTronGPT-20B, openchat__3.5, vicuna-7b, vicuna-13b, vicuna-33b, WizardLM-13b, zephyr-7b, DeepSeek R1, 2-Shot H+Expl SDoH-GPT, 0-Shot SDoH-GPT, 2-Shot E SDoH-GPT
Encoder Decoder Foundation Models	T5-2sQA, T5-Event, T5-large seq2seq, T5 v1.1 large (seq2seq), T5 v1.1 large (SHACM→SHACW), T5 v1.1 large (SHACM+SHACW), Fine-tuned Flan-T5 XL, Fine-tuned Flan-T5 XXL, Flan-T5, Flan-T5 Large, Flan-T5 XL, Flan-T5 XL + Semantic Type, Flan-T5 XL (gold data only), Flan-T5 XL (with synthetic data), Flan-T5 XXL (with synthetic data), Flan-T5 XXL 0-shot, Flan-T5 XXL 5-shot, Flan-T5 XXL finetuned on ANLI, Flan-T5 XXL finetuned on SDOH-NLI, Flan-T5 XXL finetuned on ANLI + SDOH-NLI, Flan-UL2 0-shot, Flan-UL2 5-shot, Google/flan-ul2 (zero-shot), Google/flan-ul2 (2-shot), Sentence-T5 11B, Flan-T5 (fine-tuned), FLAN-T5-XL, Microsoft (T5)
Parameter Efficient Fine Tuning	GatorTron-345M-Ptuning, GatorTron-3.9B-Ptuning, GatorTron-8.9B-Ptuning, GatorTronGPT-5B-Ptuning, GatorTronGPT-20B-Ptuning, GatorTron-3.9B (soft prompt, unfrozen), GatorTron-8.9B (soft prompt, unfrozen), GatorTron-345M (soft prompt, unfrozen), GatorTron-3.9B (soft prompt, frozen), GatorTron-8.9B (soft prompt, frozen), BERT (no prompt, unfrozen), BERT-base (gold data only)
Transformer Hybrid Architectures	BERT-CNN, BERT-BiGRU, BiLSTM-CRF, BiLSTM-CRF-MTL, BiLSTM-CNN-CRF (Proposed), BNP (BiLSTM-CNN-CRF), Event Extractor (Bio+Discharge Summary BERT + bi-LSTM + CRF), Surrogate Classifier (Bio+Discharge Summary BERT + bi-LSTM), MLTB (Multilabel Multitask BERT), mSpERT, mSpERT (Experiment 1), mSpERT (Experiment 2), mSpERT (Experiment 3), mSpERT (fine-tuned), T5-3B + RoBERTa Joint Trigger and Argument, T5-3B + RoBERTa Overlap Trigger + Overlap Argument, BioBERT + RoBERTa Joint Trigger and Argument, Flair + RoBERTa Joint Trigger and Argument, Triaffine Independent Trigger + Argument, Triaffine Joint Trigger + Argument, ClinicalBERT with Decision Tree, ClinicalBERT with Random Forest
RNN LSTM Models	BiLSTM, LSTM, LSTM__general, LSTM__mimic, LSTM with word2vec, CNN+LSTM with word2vec, TLSTM, RETAIN, LSTMs, GRUs, BLSTMs, RNNs, LSTMs+Word2Vec, GRUs+Word2Vec, BLSTMs+Word2Vec, RNNs+Word2Vec, LSTMs+GloVe, GRUs+GloVe, BLSTMs+GloVe, RNNs+GloVe, RNN
CNN Models	CNN, CNN with word2vec, CNN NER, spaCy's CNN-based NER, SWCNN with 200-dimensional word2vec, SWCNN with 100-dimensional word2vec, CNNs, CNNs+Word2Vec, CNNs+GloVe, Shallow Neural Network, CNN NER

Continued on next page

Table S4 – continued from previous page

Category	Methods
Embedding Models	word2vec, word2vec (context size = 5), word2vec (context size = 15), Sentence Transformer all-mpnet-base-v2, GTR XXL, Sentence-BERT, Word2Vec with Decision Tree, Word2Vec with SVC, Word2Vec with Random Forest
Classical ML	Logistic Regression, Logistic Regression (LASSO), Regularized logistic regression, Logistic Regression with Keywords, Linear SVM, Linear SVM with Bag of Words, Random Forest, Random Forest using n-gram, SVM, Support-vector machine-based classifier, Conditional random field classifier, XGBoost, XGBoost trained on 2-Shot H+Expl SDoH-GPT annotations, XGBoost trained on 0-Shot SDoH-GPT annotations, XGBoost trained on 2-Shot E SDoH-GPT annotations, XGBoost trained on 2-Shot H SDoH-GPT annotations, NB+TF-IDF, SVM+TF-IDF, RF+TF-IDF, LR+TF-IDF, DT+TF-IDF, LR, DT, RF
Rule Based Systems	Rule-based NLP algorithm, Rule-based Pipeline, Rule-based approaches only, Rule-based NLP System, NLP application (rule-based), General Architecture for Text Engineering, Moonstone (Rule-based NLP system), Moonstone, EasyCIE Rules-based NLP Engine, Rule based, NLP4Eye (Rule-based), Rule-based System
Clinical NLP Toolkits	cTAKES, cTAKES (Default), cTAKES (InfoCommons), cTAKES (SDoH), Yale cTAKES extension (YTEX), CLAMP, MetaMap, BioMedICUS, OHNLP Toolkit with MedTagger, NimbleMiner, Linguamatics I2E version 5.3, ezDI, Spark NLP, JSL NER (ner_sdoh_en), Refined spaCy PhraseMatcher NLP Algorithm, EntityRuler module of spaCy 2.3 Python toolkit, NegEx, UMLS, Keyword Processor
Hybrid Ensemble Methods	Combined machine-learning and rule-based approaches, Hybrid NLP + ML (XGBoost), Hybrid (Pattern Matching + Logistic Regression), RoBERTa-Ensemble Classification, Structured EHR + Text, NLP + ICD10CM Combined Approach, Multi-stage LLM Framework
Specialized Task Specific Models	BERT-MRC, BERT-MIMIC-MRC, RoBERTa-MIMIC-MRC, GatorTron-MRC, RoBERTa-MIMIC-Trial, Note-based (NB) Model, Terminology-based (TB) Model, ReHouSED (NLP-derived measure), SENTLI, SENTLI, threshold tuned, CAML, LSFA, KEPT, Sleep Phenotyping NLP Algorithm, Housing Status NLP Algorithm, NLP-CAM, Pattern Matching Baseline, FIIESCT (Food Insecurity in Electronic and Social Work Texts), Proposed Approach (Context-Specific Feature Augmentation), LSI, WNLing (WordNet + Linguistic)
Baseline Reference Models	Baseline Model (Structured EHR Only), Baseline + NLP Keyword Model, Baseline + NLP Topic Model (LDA), Baseline (Domain Expert), Manual Chart Review, Structured Data Field, Text Only, Structured EHR Only, ICD-10 Code, ICD Diagnosis Codes, ICD10CM-based Classification, Substance Use - Text Only, Bag of Words (BOW), TF-IDF weighted cosine similarity retrieval, Word2vec-based Information Retrieval with TF-IDF, Keyword Search, Lexical association (Chi-square test), RegEx (Regular Expression), RegEx, Deep learning algorithm, Transformer-based NLP model (BERT/GPT), Transformer-based NLP model, ULisboa, KEEPHA, CMU, Yale, UWisc

D Summary of studies

Table S5 shows the papers reviewed in this study along with some summarized data items.

Table S5: Summary of SDOH NLP studies.

Ref. Year Type	NCR Score	Research Question	Dataset	SDOH Type
[1] 2016 Conference	1	Automated vocabulary generation methods to improve NLP-based extraction of substance abuse mentions from psychiatric notes	MIMIC-II, University of Utah Psychiatry Notes	Individual
[2] 2017 Journal	0.36	Text mining pipeline to process unstructured clinical notes from psychiatric evaluations and extract clinical and social parameters (e.g., age, gender, history of alcohol use, violent behaviors)	CEGS N-GRID 2016 Shared Task (Partners Healthcare / Harvard Medical School)	Individual
[3] 2017 Conference	1.64	How social history documentation in EHRs varies by age and demographics using manual and automated topic analysis	Fairview Health Services Clinical Data Repository (CDR) (Private EHR dataset)	Individual
[4] 2018 Journal	0.93	Can DL be used to automatically classify social media posts into critical (requiring urgent intervention) and uncritical categories for DV crisis services?	Facebook	Individual
[5] 2018 Journal	1.87	Assess the prevalence of seven social risk factors from physician notes compared to claims and structured EHR data and their association with 30-day hospital readmissions	Partners Healthcare System HER	Individual
[6] 2018 Journal	1.14	NLP to extract homelessness and adverse childhood experiences from a large EHR repository	Vanderbilt Synthetic Derivative Database (Deidentified EHR dataset)	Individual
[7] 2018 Conference	0.21	Method to define collective social attitudes (racism, homophobia) from Twitter posts to assess exposure geography beyond administrative boundaries	Twitter API (geo-located tweets from NYC)	Structural
[8] 2018 Conference	0.41	Develop a gold-standard corpus and apply semi-supervised learning to extract SBDH related to sexual health from clinical documentation	CUIMC Clinical Data Warehouse (Private EHR dataset)	Individual
[9] 2018 Journal	1.44	Can NLP improve predictive models for identifying individuals at elevated risk of HIV infection using information from clinical records?	CDW at New York Presbyterian Hospital—Columbia University Medical Center	Individual
[10] 2019 Conference	0.13	DL pipeline to classify life events from tweets, using job loss as a use case	Twitter data	Individual
[11] 2019 Journal	2.55	Examine characteristics of SBDH data captured in EHRs of a multilevel academic healthcare system	Johns Hopkins IDR	Both
[12] 2019 Journal	1.16	NLP to identify social isolation in prostate cancer patients using clinical narratives	MUSC Research Data Warehouse	Individual

Continued on next page

Table S5 – Continued from previous page

Ref. Year Type	NCR Score	Research Question	Dataset	SDOH Type
[13] 2019 Journal	1.78	Construct a novel gold standard dataset from social media with multi-class annotation	DV Dataset	Individual
[14] 2019 Conference	0.53	NLP pipeline to ascertain marital status from structured and unstructured healthcare data	University of Utah EDW	Individual
[15] 2019 Journal	0.28	Predictive model for 5-year kidney allograft survival using EHR with NLP	UNOS database, EHR data from MUSC, Transplant database (Velos)	Individual
[16] 2019 Conference	1.11	NLP to identify patients with significant social determinant-related problems from EHRs focusing on four psychosocial vital signs	EHR from hospitals and clinics in Oregon	Individual
[17] 2019 Journal	0.38	Compare sensitivity and specificity of self-reported incarceration vs. various EHR-based methods (administrative data, reentry services, NLP)	Veterans Aging Cohort Study (VACS)	Individual
[18] 2019 Journal	1.52	Develop an open-source rule-based NLP system to extract social risk factors (housing situation, living alone, social support) from clinical notes	VHA EHR notes	Individual
[19] 2019 Journal	0.56	Association between medical imaging utilization and socioeconomic, demographic, and clinical factors in pediatric ED visits using NLP-enhanced predictive models	National Hospital Ambulatory Medical Care Survey (NHAMCS-ED, 2012–2016)	Individual
[20] 2020 Journal	0.08	NLP approach to identify financial stress from clinical narratives of prostate cancer patients	Research Data Warehouse (RDW)	Individual
[21] 2020 Journal	1.25	NLP system to infer SBDOH and patient risk status using structured and unstructured EHR data	Columbia University Medical Center HER	Individual
[22] 2020 Journal	0.34	Framework to mine, evaluate, and recommend new SDOH-related concepts using word embeddings trained on unstructured data	Wikipedia corpus extracted via DBpedia	Structural
[23] 2020 Journal	0.24	NLP pipeline to identify SDOH trends and their relationships with health outcomes during COVID-19 using knowledge graphs and population trend data	Google Trends (public SDOH-related search data), PubMed research corpus (processed via UMLS and MetaMap)	Structural
[24] 2020 Journal	3.51	Analyze Voluntary National Reviews (VNRs) and identify country-specific sustainable development strategies using text mining	VNRs dataset	Structural
[25] 2020 Journal	0.58	Evolution of social health research topics using Latent Dirichlet Allocation (LDA) topic modeling on Scopus-indexed literature	Scopus database (Query term: "social health")	Both
[26] 2021 Journal	0.44	Summarize SDOH barriers and implications for low HPV vaccination rates among young adults (18-26 years)	Multiple	Both
[27] 2021 Conference	1.44	Extract SBDOH from clinical narratives using transformer-based NLP models and compare with structured EHR data	UF Health IDR	Both
[28] 2021 Journal	1.19	Deploy a Moonstone NLP system within Vanderbilt University Medical Center to identify SDOH	VUMC EHRs system	Both
[29] 2021 Journal	2.8	Annotated corpus for SDOH extraction, use active learning to enhance annotation efficiency, and implement a neural event extraction model for SDOH	MIMIC-III, University of Washington Clinical Dataset	Individual
[30] 2021 Journal	0.3	To measure the proportion of older hospitalized veterans with five key SDOH as documented in administrative data.	VHA admin data	Individual
[31] 2021 Journal	1.58	Investigate how to leverage clinical notes using multi-label learning to classify SDOH in mental health and substance use disorder patients	Clinical data warehouse at University of North Carolina Health System	Both
[32] 2021 Conference	0	Understand documentation practices of stressful life events in clinical reports generated outside of mental health specialties and evaluate existing NLP systems for detecting stress-related concepts	MHealthFairview enterprise EHR dataset	Individual
[33] 2021 Journal	0.55	Text mining approach in identifying phrases referring to housing issues in EHR free-text notes and assess demographic/clinical characteristics of patients with housing issues	De-identified EHR data from a New England medical group	Individual
[34] 2021 Journal	2.77	Predict first-time suicide attempts using a large data-driven approach applying NLP and ML to unstructured clinical notes and structured EHRs data	UPMC's Medical ARchival System	Both
[35] 2021 Journal	0.44	What are the individual and co-occurring social needs that lead to referrals to social workers in primary care	EHRs from Eskenazi Health and data from the Indiana Network for Patient Care (INPC)	Both
[36] 2021 Journal	0.91	To develop, evaluate and implement a novel NLP-based measure of Veteran housing stability	VA CDW	Individual

Continued on next page

Table S5 – Continued from previous page

Ref. Year Type	NCR Score	Research Question	Dataset	SDOH Type
[37] 2021 Journal	0.55	Examine the usefulness of NLP for SBDH extraction from unstructured EHR text	MIMIC-III	Both
[38] 2021 Conference	0.08	Leverage knowledge graphs and population trends to analyze SDOH related to COVID-19 using PubMed literature and Google Trends	Google Trends, PubMed abstracts	Structural
[39] 2021 Journal	0.91	To develop, evaluate and implement a novel application using NLP to text mine occupations from the free-text of psychiatric clinical notes.	Clinical Record Interactive Search (CRIS) platform	Individual
[40] 2022 Journal	0.72	NLP to extract life event indicators from clinical notes to improve the identification of acute risk factors for suicide	EHRs from the VA CDW, death records from the NDI	Both
[41] 2022 Journal	1.02	Assess the documentation of SDOH for lung cancer patients in clinical narratives	UF Health IDR	Both
[42] 2022 Journal	0.08	Investigate feasibility of behavioral predictive analytics to optimize patient engagement in diabetes self-management	NA	Both
[43] 2022 Journal	0.79	Develop annotation schema to deeply characterize OUD, and automate the schema using ML and DL-based approaches	MIMIC-III	Both
[44] 2022 Journal	5.18	Can deep neural networks (trained on MIMIC-III) extract SDOH when SDOH categories are systematically defined using standard biomedical and psychiatric ontologies (SNOMED-CT and DSM-IV)?	MIMIC-III	Individual
[45] 2022 Journal	0.49	NER to extract both clinical and non-clinical risk factors related to COVID-19 from biomedical texts	COVID-19 Biomedical NER Dataset, derived from LitCOVID and PubMed articles	Individual
[46] 2022 Conference	0.45	Evaluate a ML pipeline to detect biomedical named entities (both clinical and non-clinical) in COVID-19 texts	COVID-19 case reports dataset (own developed), and JNLPBA, NCBI-Disease, BC5CDR, BC2GM (benchmark datasets)	Both
[47] 2022 Journal	1.21	Evaluate if NLP algorithm could be adapted to extract markers of residential instability from EHRs of 3 healthcare systems	De-identified EHR data from JHHS, JPMAS, KPSC	Individual
[48] 2022 Journal	1.02	NLP approach to identify SDOH from digitized free-text provider notes	YCLS Survey Records and Provider Notes from KPMAS EHR (HealthConnect)	Individual
[49] 2022 Journal	1.32	Improve ascertainment of two suicidal outcomes—suicidal ideation and suicide attempt—from EHR data using scalable NLP.	VUMC HER	Individual
[50] 2022 Journal	0.3	NLP model that can identify pediatric patients exposed to parental justice involvement (prison, jail, parole, probation) within unstructured clinician notes in a large pediatric HER	EHRs data	Individual
[51] 2022 Journal	0.72	Extract psychosocial concepts from chart notes using NLP and predict their impact on healthcare utilization for patients with multimorbidity	NA	Both
[52] 2022 Journal	0.45	Compare active (manual) vs. passive (NLP-based) screening methods for identifying SDOH	EHR Data from a midwestern tertiary referral hospital system	Both
[53] 2022 Journal	0.23	Extract social factors from clinical notes using common text classification methods	MIMIC-III	Both
[54] 2023 Journal	0.46	Develop a multi-stage NLP pipeline for automatically extracting SDOH data from clinical notes.	SHAC	Both
[55] 2023 Journal	1.22	NLP model to extract SDOH-related circumstances and suicide crises from death investigation narratives.	NVDRS	Individual
[56] 2023 Journal	0.64	Develop rule-based model for identifying three major domains of social needs (residential instability, food insecurity, and transportation) from unstructured data in EHRs	EHR from Johns Hopkins Health System	Individual
[57] 2023 Journal	0.23	Evaluate the accuracy of different data sources in assessing longitudinal housing outcomes and compare against structured EHR (ICD-10 codes, administrative records)	VA Greater Los Angeles Healthcare System (EHR), VA's CDW & HOMES, Patient-Reported Housing History via TLFB Inventory	Individual
[58] 2023 Journal	2.49	Investigate associations between SDOH and suicide deaths among US veterans	EHR database from the VHA CDW	Both
[59] 2023 Conference	0.64	Improve Wellness Dimension (WD) classification in Reddit posts through data augmentation using generative NLP	Wellness Dimensions Dataset	Individual
[60] 2023 Journal	0.64	Develop an NLP model to detect eviction status from EHR notes in the VHA system	VHA CDW	Individual

Continued on next page

Table S5 – Continued from previous page

Ref. Year Type	NCR Score	Research Question	Dataset	SDOH Type
[61] 2023 Journal	2.26	Develop NLP methods to extract SDOH from clinical notes of chronic lower back pain (cLBP) patients	UCSF cLBP Clinical Notes Dataset	Both
[62] 2023 Conference	0.35	Benchmark 7 pre-trained transformer-based language models for recognizing SDOH terms from clinical notes	SBDH-MIMIC Annotated Corpus	Both
[63] 2023 Journal	2.37	NLP system to extract clinical concepts and relations with good generalizability for cross-institution applications	2018 n2c2 dataset (drug-ADE dataset), 2022 n2c2 dataset (SDOH dataset)	Individual
[64] 2023 Journal	0.81	NLP algorithm using ML to identify and classify documentation of preoperative cannabis use in unstructured clinical notes	PRECEDE Bank, UF Health IDR, MIMIC-III	Individual
[65] 2023 Journal	0.06	Impact of SDOH on students' perception of COVID-19 social distancing, mental health, and quality of life	Korean Undergraduate COVID-19 Social Distancing Survey	Structural
[66] 2023 Journal	1.68	Can the prediction accuracy of a ML model for postdischarge suicide risk be improved by incorporating information from clinical notes and public records?	VHA CDW, VA Suicide Prevention Applications Network, Geospatial SDOH database assembled from diverse govt sources, LexisNexis SDOH database	Individual
[67] 2023 Journal	0.58	Identify and compare evidence of housing instability using three different data sources: structured diagnosis codes, semi-structured addresses, and unstructured clinical notes in populations with substance use disorders.	EHR from the University of Kentucky HealthCare network	Both
[68] 2023 Journal	1.74	NLP system to extract SDOH events from clinical notes	MIMIC-III,SHAC,University of Washington and Harborview Medical Centers (UW) notes	Individual
[69] 2023 Journal	1.56	Propose 2 DL approaches to the extraction of SDOH from clinical text that the authors evaluate in the context of the National NLP Clinical Challenges (n2c2) Track 2 challenge	SDOH-N2C2 Dataset, derived from MIMIC-III, SHAC, In-house corpus, NO-SDOH corpus	Individual
[70] 2023 Journal	0.12	Assess prevalence of unmet social needs in Medicare enrollees with multiple chronic illnesses enrolled in care management services using NLP	Free text notes from Medicare Chronic Care Management patient encounters	Both
[71] 2023 Journal	0.87	Report on generalizability of NLP to extract individual social factors from clinical notes	EHR from University of Florida and Indiana University Patients	Individual
[72] 2023 Conference	0.12	NLP system to extract SDOH data from unstructured clinical notes to help Emergency Department social workers identify patients needing SDOH support	NorthShore-Edward-Elmhurst Health EDW	Both
[73] 2023 Journal	1.39	How did the discourse in mental health-related subreddit communities evolve between 2019 and 2021	r/Depression and r/Anxiety	Individual
[74] 2023 Journal	2.32	DL-based NLP to extract detailed SDOH information from EHRs	SHAC (model training), University of Washington Medicine EHR	Individual
[75] 2023 Journal	1.68	NLP algorithm that identifies SDOH from unstructured EHRs for patients with ADRD	Emergency department and inpatient social worker notes from Michigan Medicine EHR database	Individual
[76] 2023 Journal	1.5	NLP algorithms to extract the presence of social factors from clinical text in housing, financial, and unemployment.	Indiana Network for Patient Care (INPC) Clinical Notes Dataset	Both
[77] 2023 Journal	0.46	Assess differences between respondents and those refusing participation in a social factor screening study conducted in an emergency department	Clinical notes and EHR data from Eskenazi Health	Both
[78] 2023 Conference	0.41	How to predict LGBTQ+ minority stress using neural networks, focusing on linguistically sophisticated social determinants of health disparities	LGBTQ+ Minority Stress on Social Media (MiSSoM+) dataset	Both
[79] 2023 Conference	1.27	Explore the use of GPT-4 for extracting SDOH from clinical notes using one-shot prompting.	SHAC, MIMIC-III, University of Washington Harborview Medical Centers (UW)	Individual
[80] 2023 Conference	0.29	Introduce SDOH-NLI, a publicly available dataset for SDOH extraction from clinical notes	SDOH-NLI, derived from MTSamples.com (Public Transcribed Medical Reports)	Both
[81] 2023 Journal	0.69	Compare the documentation of SDOH in structured versus unstructured EHR data of patients with diabetes, and test high-throughput tools for identifying SDOH information in clinical notes.	UCSF Epic-based EHR data extracted using SQL-based deidentified Clinical Data Warehouse (De-ID CDW) and PatientExploreR.	Individual
[82] 2023 Journal	0.58	Determine the presence of SDOH information in adult EMS records and how such information is linked to other determinants by EMS personnel	2019 ESO Data Collaborative public-use research dataset.	Individual
[83] 2023 Journal	0.75	Identify social risk factors in home health care (HHC) clinical notes	Clinical notes and Outcome and Assessment Information Set (OASIS)-C2 data	Both
[84] 2023 Journal	2.78	Evaluate NLP-based information extraction techniques (rules, knowledge bases, n-grams, word embeddings, and pretrained language models) for identifying SDOH from clinical notes	Social History Annotated Corpus (SHAC)	Individual

Continued on next page

Table S5 – Continued from previous page

Ref. Year Type	NCR Score	Research Question	Dataset	SDOH Type
[85] 2023 Journal	0.46	Examine ethnic disparities in antipsychotic prescribing among individuals with psychosis in the UK	CRIS	Structural
[86] 2023 Journal	0.75	Examine the presence of SDOH in EMS clinician free text notes and quantify the association of SDOH with EMS pediatric transport decisions.	2019 ESO Data Collaborative research dataset	Both
[87] 2023 Journal	0.41	How can we identify the status of homelessness and housing instability among patients with serious illnesses using NLP?	Clinical notes from KPSC's EHR system	Both
[88] 2023 Conference	0.29	NLP to extract housing status from clinical notes in EHR data and address challenges in studying homelessness longitudinally due to irregular observations	VA CDW, HMIS, SSVF program data	Both
[89] 2023 Conference	1.16	Investigate whether a GPT model can perform zero-shot extraction of combined demographic, SDOH, and family history from de-identified clinical notes with minimal instruction.	Unnamed university hospital	Individual
[90] 2024 Conference	0.21	Investigate use of GPT for extracting addiction status (on tobacco, alcohol, and illicit substance use statuses) information from patient discharge summaries	MIMIC-III dataset.	Individual
[91] 2024 Conference	0.31	Evaluate if LLMs can conduct causal reasoning for sensing data analysis	CalEnviroScreen 4.0 database	Structural
[92] 2024 Journal	0.1	NLP on unstructured EHR text to detect and classify financial insecurity among patients with rheumatologic conditions enrolled in an iCMP	RPDR	Individual
[93] 2024 Journal	0.58	Predict the risk of ASUD in PTSD patients by analyzing lab tests, medication use, diagnosis, SDOH parameters, and psychotherapy information.	Patients from the UPMC diagnosed with PTSD	Both
[94] 2024 Journal	0.63	Evaluate epilepsy-specific LLM for intrinsic bias across demographic groups	EHR from patients who had seen an epileptologist at University of Pennsylvania Health System	Both
[95] 2024 Journal	0.31	To explore the determinants and influencing mechanisms of health-promoting behaviors in miners	Literature collected from seven databases: Google Scholar, Web of Science, PubMed Central, Scopus, Engineering Village, Cumulative Index to Nursing and Allied Health Literature, and APA PsycNet	Both
[96] 2024 Conference	0.84	How can we effectively extract SDOH from pediatric patient notes using LLMs?	PedSHAC	Both
[97] 2024 Journal	1.05	Explore capability of a unified generative LLM, GatorTronGPT, in solving seven major clinical NLP tasks using prompt tuning instead of task-specific fine-tuning	2018 n2c2 (ADE and medication extraction dataset), 2022 n2c2 (SDOH dataset), SemEval-2015, UMN abbreviation dataset, MedNLI dataset, CMED, MIMIC-III	Both
[98] 2024 Journal	0.73	Develop, train, and validate NLP techniques to more effectively identify incarceration status in EHR	collection of Emergency Department notes from the Epic EHRs system.	Individual
[99] 2024 Journal	1.26	NLP package to extract SDOH from clinical narratives and examine the bias among race and gender groups	Clinical narratives from UF Health IDR	Both
[100] 2024 Journal	0.16	How has the COVID-19 pandemic impacted the SDOH for marginalized Black and Asian communities in the US?	Reddit dataset	Both
[101] 2024 Journal	0.42	Creation of a data resource that links mental health EHRs to UK census data	CRIS from SLaM linked to the 2011 census of England and Wales	Both
[102] 2024 Conference	0	Compare NLP models for cross-domain binary relation classification, focusing on gene-disease, compound-protein, and SDOH	PubMed / MEDLINE	Both
[103] 2024 Journal	12.47	Investigate optimal methods for using LLMs to extract SDOH from EHRs, including evaluating the role of synthetic clinical text	Brigham and Women's Hospital/Dana-Farber Cancer Institute in Boston Radiotherapy clinical notes, Dana-Farber Cancer Institute Immunotherapy clinical notes, MIMIC-III	Individual
[104] 2024 Journal	3.46	Develop soft prompt-based learning architecture for LLMs and examine prompt-tuning using frozen/unfrozen LLMs	2018 n2c2 dataset (track 2), 2022 n2c2 dataset (track 2), MIMIC-III, University of Washington clinical notes	Individual
[105] 2024 Journal	0.1	Create novel analysis-to-visualization methods to find associations between county-level social determinants	County-level mortality rates from CDC WONDER and social determinants data from County Health Rankings.	Both
[106] 2024 Conference	0.16	Benchmark dataset for detecting ORAB from EHRs and classify them into nine categories	ODD (ORAB Detection Dataset)	Individual
[107] 2024 Journal	0.52	Is poverty associated with suicidal ideation among Hispanic mental healthcare patients from 2016 to 2019?	Holmusk data from MindLinc EHR system	Both

Continued on next page

Table S5 – Continued from previous page

Ref. Year Type	NCR Score	Research Question	Dataset	SDOH Type
[108] 2024 Journal	0.52	Develop a question-answering (QA) framework to extract information about injection drug use (IDU) from free-text clinical notes in EHRs.	VA CDW	Individual
[109] 2024 Journal	0.05	Evaluate how adding new SDOH terms to the Medical Subject Headings (MeSH) thesaurus would affect PubMed's automatic term mapping (ATM)	PubMed / MEDLINE	Both
[110] 2024 Journal	0.21	Lexicon of housing-related concepts and rule-based NLP methods for identifying housing-related concepts within clinical text	ChatGPT-generated emergency department notes SUD cohort documents HEC cohort documents	Structural
[111] 2024 Journal	0.68	What is the potential of social work notes as a source of data on SDOH compared to physician notes?	UCSF DeID CDW-OMOP	Both
[112] 2024 Journal	0.31	Investigate sociodemographic, clinical, and service use variables associated with unemployment for service users with SMI	EHRs from SLaM NHS Trust accessed via CRIS	Both
[113] 2024 Journal	0.68	Explore the relationship between human sentiment on social media (tweets), urban building characteristics, and socio-spatial dynamics of NYC boroughs, with focus on Energy Bill and HVAC terms	X (Twitter) dataset, NYC PLUTO (Primary Land Use Tax Lot Output) dataset, Low-income Energy Affordability Data (LEAD) Tool	Both
[114] 2024 Journal	5.03	Framework that represents human lives in a way similar to language and adapt NLP techniques to examine the evolution and predictability of human lives based on detailed event sequences.	Labour Market Account (AMRUN), National Patient Registry (LPR), POSAP Study (for personality data)	Both
[115] 2024 Conference	0.21	Multilingual conversational AI agent for SDOH surveys and evaluate LLMs to map voice responses to structured survey answers	Custom Audio Dataset	Both
[116] 2024 Journal	0.84	Feasibility of using LLMs to automate the extraction of assessment criteria and risk factors from preventive healthcare guidelines	USPSTF published preventive care guidelines rated as "A" and "B" applicable to adults	Both
[117] 2024 Journal	0.73	LLM classifier for identifying SDOH from unstructured clinical notes using synthetic datasets generated by GPT	i2b2 (2014 Deidentification and Heart Disease dataset) MIMIC-III (Medical Information Mart for Intensive Care-III) Institutional EHR data from UCSD	Individual
[118] 2024 Conference	0.05	To augment EHR discharge summaries with context-specific semantic knowledge from biomedical literature to generate feature representations needed for accurate SDOH extraction	MIMIC-IV-SDoH	Individual
[119] 2024 Conference	0.16	SDOH information extraction from EHRs for imbalanced SDOH categories due to data scarcity issues.	MIMIC-III SDOH	Individual
[120] 2024 Journal	0.37	Extract SDOH (language barriers, living alone, employment status, and education) from inpatient EMR data using NLP compatible with current EMR systems	Data from Allscripts Sunrise Clinical Manager EMR system	Individual
[121] 2024 Journal	0.31	NLP for identifying patients' unmet social needs to enable timely intervention	Mayo Clnic EHR	Individual
[122] 2024 Journal	0.58	GPT-3.5 and GPT-4 for identifying instances of both current and past housing instability from clinical notes.	Providence EHR	Individual
[123] 2024 Conference	0.73	To show efficacy of BERT and GPT in identifying and categorizing SDOH from EHRs	MIMIC	Both
[124] 2024 Journal	0.21	ML approaches for detection of SBDH from unstructured clinical notes in the EHR	MIMIC-III	Both
[125] 2025 Conference	0	Study longitudinal trajectories of food insecurity among a large cohort of food insecure Veterans	VA CDW	Individual
[126] 2025 Journal	0.55	Extract SDOH details including 17 distinct types along & various subtypes from EHRs at BC Cancer	BC Cancer oncology consultation reports	Individual
[127] 2025 Journal	1.66	Few-shot LLMs to extract SDOH from unstructured text to improve both efficiency and generalizability.	MIMIC-III (MIMIC-SBDH subset), Suicide Notes (from NVDRS), Sleep Notes (from Alzheimer's Disease dataset)	Individual
[128] 2025 Journal	5.54	Can open-source LLMs (no fine-tuning) accurately extract SDOH data from free-text clinical notes	EHR data from the Mass General Brigham (MGB) system	Individual
[129] 2025 Journal	0.28	validate a LLM-powered SBDH-Reader to extract structured SBDH data from clinical notes through prompt engineering	"MIMIC-III database (with two subsets: MIMIC-G and MIMIC-A) UTSW dataset (heart failure cohort)	" Individual
[130] 2025 Journal	1.11	Develop generalizable NLP to extract SDOH information from clinical notes	"HCPC (UTHealth Harris County Psychiatric Center) UTP (UT Physicians) MIMIC-III Mayo	" Individual
[131] 2025 Journal	0.55	Assess cTAKES on EHR to ascertain housing and food insecurity among older adults, and validate performance via manual chart review	UCSF Health EHRs	Individual

Continued on next page

Table S5 – Continued from previous page

Ref. Year Type	NCR Score	Research Question	Dataset	SDOH Type
[132] 2025 Journal	4.43	validate NLP to extract social support (SS) and social isolation (SI) from psychiatric encounter notes at two health systems	EHR clinical notes from the MSHS data warehouse and the WCM enterprise data warehouse (psychiatric encounters).	Individual
[133] 2025 Conference	0	Transfer learning ability of generative LLMsfor cross institution and cross-disease clinical NLP applications through P-tuning	2022 n2c2 challenge and a cross-disease dataset from the University of Florida (UF) Health	Individual
[134] 2025 Conference	0	Do LLMs exhibit spurious correlations in SDOH extraction? Do in-context learning and fine-tuning reduce these correlations?	MIMIC SHAC	Individual
[135] 2025 Journal	1.94	LLM to extract stressful life events from social history sections of clinical notes and its impact on colorectal cancer.	Medical University of South Carolina (EHR in Epic)	Individual
[136] 2025 Conference	1.38	LLMs & NLP to mine SDoH from literature and integrate it with AD-related biological entities from PrimeKG	PubMed literature (abstracts) and PrimeKG (a general-purpose knowledge graph for precision medicine)	Both
[137] 2025 Conference	0.28	LLMs to extract SDOH from clinical text and analyze their association with heart failure patient 30-day readmissions	MIMIC-III	Individual
[138] 2025 Journal	0	Assess the association between NLP-derived SDoH factors and pregnancy complications	MIMIC-III MIMIC-IV	Individual
[139] 2025 Journal	0	LLM to extract suicide-related SDOH from unstructured text and analyzing pivotal stressors preceding suicide incidents	NVDRS	Individual
[140] 2025 Journal	0	NLP-derived clinical information accessible and queryable across the ENACT network	ENACT network, which connects EHR data repositories across 57 Clinical and Translational Science Awards (CTSA) hubs.	Individual
[141] 2025 Journal	0	Identify transportation insecurity in free-text ophthalmology clinic notes using NLP	EHR from UPMC Department of Ophthalmology	Both
[142] 2025 Journal	0.28	NLP to extract SDOH from free-text clinician notes and quantify the association between SDOH and access to the transplant waitlist	Duke University Health System	Individual

References

[1] S. Velupillai, D. Mowery, M. Conway, J. Hurdle, and B. Kious. Vocabulary development to support information extraction of substance abuse from psychiatry notes. pages 92–101. Association for Computational Linguistics (ACL), 2016. doi:10.18653/v1/W16-2912.

[2] Dai HJ, Su EC, Uddin M, Jonnagaddala J, Wu CS, and Syed-Abdul S. Exploring associations of clinical and social parameters with violent behaviors among psychiatric patients. *J Biomed Inform*, 75S:S149–S159, 2017. doi:10.1016/j.jbi.2017.08.009. Place: United States.

[3] E.A. Lindemann, E.S. Chen, Y. Wang, S.J. Skube, and G.B. Melton. Representation of Social History Factors Across Age Groups: A Topic Analysis of Free-Text Social Documentation. *AMIA Annu Symp Proc*, 2017:1169–1178, 2017. PMID: 29854185.

[4] S. Subramani, H. Wang, H. Q. Vu, and G. Li. Domestic Violence Crisis Identification From Facebook Posts Based on Deep Learning. *IEEE Access*, 6:54075–54085, 2018. doi:10.1109/ACCESS.2018.2871446.

[5] A.S. Navathe, F. Zhong, V.J. Lei, F.Y. Chang, M. Sordo, M. Topaz, S.B. Navathe, R.A. Rocha, and L. Zhou. Hospital Readmission and Social Risk Factors Identified from Physician Notes. *Health Serv. Res.*, 53(2):1110–1136, 2018. doi:10.1111/1475-6773.12670. Publisher: Blackwell Publishing Inc.

[6] C.A. Bejan, J. Angiolillo, D. Conway, R. Nash, J.K. Shirey-Rice, L. Lipworth, R.M. Cronin, J. Pulley, S. Kripalani, S. Barkin, K.B. Johnson, and J.C. Denny. Mining 100 million notes to find homelessness and adverse childhood experiences: 2 case studies of rare and severe social determinants of health in electronic health records. *J. Am. Med. Informatics Assoc.*, 25(1):61–71, 2018. doi:10.1093/jamia/ocx059. Publisher: Oxford University Press.

[7] K. Relia, M. Akbari, D. Duncan, and R. Chunara. Socio-spatial self-organizing maps: Using social media to assess relevant geographies for exposure to social processes. *Proc. ACM Hum. Comput. Interact.*, 2(CSCW), 2018. doi:10.1145/3274414. Publisher: Association for Computing Machinery.

[8] D.J. Feller, J. Zucker, O.B. Don't Walk, B. Srikishan, R. Martinez, H. Evans, M.T. Yin, P. Gordon, and N. Elhadad. Towards the Inference of Social and Behavioral Determinants of Sexual Health: Development of a Gold-Standard Corpus with Semi-Supervised Learning. *AMIA Annu Symp Proc*, 2018:422–429, 2018. PMID: 30815082.

[9] Daniel J. Feller, Jason Zucker, Michael T. Yin, Peter Gordon, and Noémie Elhadad. Using Clinical Notes and Natural Language Processing for Automated HIV Risk Assessment. *JAIDS: Journal of Acquired Immune Deficiency Syndromes*, 77(2):160–166, 2018. doi:10.1097/QAI.0000000000001580.

[10] X. Du, J. Bian, and M. Prosperi. An operational deep learning pipeline for classifying life events from individual tweets. volume 898, pages 54–66. Springer Verlag, 2019. doi:10.1007/978-3-030-11680-4_7.

[11] E. Hatef, M. Rouhizadeh, I. Tia, E. Lasser, F. Hill-Briggs, J. Marsteller, and H. Kharrazi. Assessing the availability of data on social and behavioral determinants in structured and unstructured electronic health records: A retrospective analysis of a multilevel health care system. *JMIR Med. Inform.*, 7(3), 2019. doi:10.2196/13802. Publisher: JMIR Publications Inc.

[12] V.J. Zhu, L.A. Lenert, B.E. Bunnell, J.S. Obeid, M. Jefferson, and C.A. Hughes-Halbert. Automatically identifying social isolation from clinical narratives for patients with prostate Cancer. *BMC Med. Informatics Decis. Mak.*, 19(1), 2019. doi:10.1186/s12911-019-0795-y. Publisher: BioMed Central Ltd.

[13] S. Subramani, S. Michalska, H. Wang, J. Du, Y. Zhang, and H. Shakeel. Deep Learning for Multi-Class Identification From Domestic Violence Online Posts. *IEEE Access*, 7:46210–46224, 2019. doi:10.1109/ACCESS.2019.2908827.

[14] B.T. Bucher, J. Shi, R.J. Pettit, J. Ferraro, W.W. Chapman, and A. Gundlapalli. Determination of Marital Status of Patients from Structured and Unstructured Electronic Healthcare Data. *AMIA Annu Symp Proc*, 2019:267–274, 2019. PMID: 32308819.

[15] D.A. DuBay, Z. Su, T.A. Morinelli, P. Baliga, V. Rohan, J. Bian, D. Northrup, N. Pilch, V. Rao, T.R. Srinivas, P.D. Mauldin, and D.J. Taber. Development and future deployment of a 5 years allograft survival model for kidney transplantation. *Nephrology*, 24(8):855–862, 2019. doi:10.1111/nep.13488. Publisher: Blackwell Publishing.

[16] D. Dorr, C.A. Bejan, C. Pizzimenti, S. Singh, M. Storer, and A. Quinones. Identifying patients with significant problems related to social determinants of health with natural language processing. volume 264, pages 1456–1457. IOS Press, 2019. doi:10.3233/SHTI190482.

[17] Wang EA, Long JB, McGinnis KA, Wang KH, Wildeman CJ, Kim C, Bucklen KB, Fiellin DA, Bates J, Brandt C, and Justice AC. Measuring Exposure to Incarceration Using the Electronic Health Record. *Med Care*, 57 Suppl 6 Suppl 2(Suppl 6 2):S157–S163, 2019. doi:10.1097/MLR.0000000000001049. Place: United States.

[18] M. Conway, S. Keyhani, L. Christensen, B.R. South, M. Vali, L.C. Walter, D.L. Mowery, S. Abdelrahman, and W.W. Chapman. Moonstone: A novel natural language processing system for inferring social risk from clinical narratives. *J. Biomed. Semant.*, 10(1), 2019. doi:10.1186/s13326-019-0198-0. Publisher: BioMed Central Ltd.

[19] X. Zhang, M.F. Bellolio, P. Medrano-Gracia, K. Werys, S. Yang, and P. Mahajan. Use of natural language processing to improve predictive models for imaging utilization in children presenting to the emergency department. *BMC Med. Informatics Decis. Mak.*, 19(1), 2019. doi:10.1186/s12911-019-1006-6. Publisher: BioMed Central Ltd.

[20] Zhu V, Lenert L, Bunnell B, Obeid J, Jefferson M, and Halbert CH. Automatically Identifying Financial Stress Information from Clinical Notes for Patients with Prostate Cancer. *Cancer Res Rep*, 1(1), 2020. doi:10.61545/crr-1-102. Place: Canada.

[21] D.J. Feller, O.J. Bear Don't Walk Iv, J. Zucker, M.T. Yin, P. Gordon, and N. Elhadad. Detecting Social and Behavioral Determinants of Health with Structured and Free-Text Clinical Data. *Appl. Clin. Informatics*, 11(1):172–181, 2020. doi:10.1055/s-0040-1702214. Publisher: Georg Thieme Verlag.

[22] J.H. Bettencourt-Silva, N. Mulligan, M. Sbodio, J. Segrave-Daly, R. Williams, V. Lopez, and C. Alzate. Discovering new social determinants of health concepts from unstructured data: Framework and evaluation. volume 270, pages 173–177. IOS Press, 2020. doi:10.3233/SHTI200145.

[23] J.H. Bettencourt-Silva, N. Mulligan, C. Jochim, N. Yadav, W. Sedlazek, V. Lopez, and M. Gleize. Exploring the social drivers of health during a pandemic: Leveraging knowledge graphs and population trends in COVID-19. volume 275, pages 6–11. IOS Press BV, 2020. doi:10.3233/SHTI200684.

[24] V. Sebestyén, E. Domokos, and J. Abonyi. Focal points for sustainable development strategies—Text mining-based comparative analysis of voluntary national reviews. *J. Environ. Manage.*, 263, 2020. doi:10.1016/j.jenvman.2020.110414. Publisher: Academic Press.

[25] S.M. Cho, C.-U. Park, and M. Song. The evolution of social health research topics: A data-driven analysis. *Soc. Sci. Med.*, 265, 2020. doi:10.1016/j.socscimed.2020.113299. Publisher: Elsevier Ltd.

[26] O.A. Olusanya, N. Ammar, R.L. Davis, R.A. Bednarczyk, and A. Shaban-Nejad. A Digital Personal Health Library for Enabling Precision Health Promotion to Prevent Human Papilloma Virus-Associated Cancers. *Front. Digit. Health*, 3, 2021. doi:10.3389/fdgth.2021.683161. Publisher: Frontiers Media SA.

[27] Z. Yu, X. Yang, C. Dang, S. Wu, P. Adekkanattu, J. Pathak, T.J. George, W.R. Hogan, Y. Guo, J. Bian, and Y. Wu. A Study of Social and Behavioral Determinants of Health in Lung Cancer Patients Using Transformers-based Natural Language Processing Models. *AMIA Annu Symp Proc*, 2021:1225–1233, 2021. PMID: 35309014.

[28] Ruth M. Reeves, Lee Christensen, Jeremiah R. Brown, Michael Conway, Maxwell Levis, Glenn T. Gobbel, Rashmee U. Shah, Christine Goodrich, Iben Ricket, Freneka Minter, Andrew Bohm, Bruce E. Bray, Michael E. Matheny, and Wendy Chapman. Adaptation of an NLP system to a new healthcare environment to identify social determinants of health. *Journal of Biomedical Informatics*, 120:103851, August 2021. ISSN 1532-0464. doi:10.1016/j.jbi.2021.103851.

[29] K. Lybarger, M. Ostendorf, and M. Yetisgen. Annotating social determinants of health using active learning, and characterizing determinants using neural event extraction. *J. Biomed. Informatics*, 113, 2021. doi:10.1016/j.jbi.2020.103631. Publisher: Academic Press Inc.

[30] Wray CM, Vali M, Walter LC, Christensen L, Abdelrahman S, Chapman W, and Keyhani S. Examining the Interfacility Variation of Social Determinants of Health in the Veterans Health Administration. *Fed Pract*, 38(1):15–19, 2021. doi:10.12788/fp.0080. Place: United States.

[31] Rachel Stemerman, Jaime Arguello, Jane Brice, Ashok Krishnamurthy, Mary Houston, and Rebecca Kitzmiller. Identification of social determinants of health using multi-label classification of electronic health record clinical notes. *JAMIA Open*, 4(3):ooaa069, July 2021. ISSN 2574-2531. doi:10.1093/jamiaopen/ooaa069.

[32] S. Datar, E.A. Lindemann, G. Silverman, R. McEwan, R. Finzel, M. Kotlyar, G.B. Melton, and S.V.S. Pakhomov. Identifying Mentions of Life Stressors in Clinical Notes. pages 153–160. Institute of Electrical and Electronics Engineers Inc., 2021. doi:10.1109/ICHI52183.2021.00033.

[33] E. Hatef, G. Singh Deol, M. Rouhizadeh, A. Li, K. Eibensteiner, C.B. Monsen, R. Bratslaver, M. Senese, and H. Kharrazi. Measuring the Value of a Practical Text Mining Approach to Identify Patients With Housing Issues in the Free-Text Notes in Electronic Health Record: Findings of a Retrospective Cohort Study. *Front. Public Health*, 9, 2021. doi:10.3389/fpubh.2021.697501. Publisher: Frontiers Media S.A.

[34] F.R. Tsui, L. Shi, V. Ruiz, N.D. Ryan, C. Biernesser, S. Iyengar, C.G. Walsh, and D.A. Brent. Natural language processing and machine learning of electronic health records for prediction of first-time suicide attempts. *JAMIA Open*, 4(1), 2021. doi:10.1093/jamiaopen/ooab011. Publisher: Oxford University Press.

[35] Abdulaziz T. Bako, Heather Walter-McCabe, Suranga N. Kasthurirathne, Paul K. Halverson, and Joshua R. Vest. Reasons for social work referrals in an urban safety-net population: A natural language processing and market basket analysis approach. *Journal of Social Service Research*, 47(3):414–425, 2021. doi:10.1080/01488376.2020.1817834.

[36] A.B. Chapman, A. Jones, A.T. Kelley, B. Jones, L. Gawron, A.E. Montgomery, T. Byrne, Y. Suo, J. Cook, W. Pettey, K. Peterson, M. Jones, and R. Nelson. ReHouSED: A novel measurement of Veteran housing stability using natural language processing. *J. Biomed. Informatics*, 122, 2021. doi:10.1016/j.jbi.2021.103903. Publisher: Academic Press Inc.

[37] A. Mitra, H. Ahsan, W. Li, W. Liu, R.D. Kerns, J. Tsai, W. Becker, D.A. Smelson, and H. Yu. Risk factors associated with nonfatal opioid overdose leading to intensive care unit admission: A cross-sectional study. *JMIR Med. Inform.*, 9(11), 2021. doi:10.2196/32851. Publisher: JMIR Publications Inc.

[38] M Gleize, N Mulligan, A Di Bari, and JH Bettencourt-Silva. Social Determinant Trends of COVID-19: An Analysis Using Knowledge Graphs from Published Evidence and Online Trends. volume 281, pages 744–748, 2021. doi:10.3233/SHTI210271.

[39] N. Chilman, X. Song, A. Roberts, E. Tolani, R. Stewart, Z. Chui, K. Birnie, L. Harber-Aschan, B. Gazard, D. Chandran, J. Sanyal, S. Hatch, A. Kolliakou, and J. Das-Munshi. Text mining occupations from the mental health electronic health record: A natural language processing approach using records from the Clinical Record Interactive Search (CRIS) platform in south London, UK. *BMJ Open*, 11(3), 2021. doi:10.1136/bmjopen-2020-042274. Publisher: BMJ Publishing Group.

[40] Destinee Morrow, Rafael Zamora-Resendiz, Jean C. Beckham, Nathan A. Kimbrel, David W. Oslin, Suzanne Tamang, and Silvia Crivelli. A case for developing domain-specific vocabularies for extracting suicide factors from healthcare notes. *Journal of Psychiatric Research*, 151: 328–338, 2022. doi:10.1016/j.jpsychires.2022.04.009.

[41] Z. Yu, X. Yang, Y. Guo, J. Bian, and Y. Wu. Assessing the Documentation of Social Determinants of Health for Lung Cancer Patients in Clinical Narratives. *Front. Public Health*, 10, 2022. doi:10.3389/fpubh.2022.778463. Publisher: Frontiers Media S.A.

[42] B. Sy, M. Wassil, H. Connelly, and A. Hassan. Behavioral Predictive Analytics Towards Personalization for Self-management: a Use Case on Linking Health-Related Social Needs. *SN COMPUT. SCI.*, 3(3), 2022. doi:10.1007/s42979-022-01092-2. Publisher: Springer.

[43] M.N. Poulsen, P.J. Freda, V. Troiani, A. Davoudi, and D.L. Mowery. Classifying Characteristics of Opioid Use Disorder From Hospital Discharge Summaries Using Natural Language Processing. *Front. Public Health*, 10, 2022. doi:10.3389/fpubh.2022.850619. Publisher: Frontiers Media S.A.

[44] SF Han, RF Zhang, LY Shi, R Richie, HX Liu, A Tseng, W Quan, N Ryan, D Brent, and FR Tsui. Classifying social determinants of health from unstructured electronic health records using deep learning-based natural language processing. *JOURNAL OF BIOMEDICAL INFORMATICS*, 127, 2022. doi:10.1016/j.jbi.2021.103984.

[45] S.R. Bashir, S. Raza, V. Kocaman, and U. Qamar. Clinical Application of Detecting COVID-19 Risks: A Natural Language Processing Approach. *Viruses*, 14(12), 2022. doi:10.3390/v14122761. Publisher: MDPI.

[46] Shaina Raza and Brian Schwartz. Detecting biomedical named entities in covid-19 texts. In *Proceedings of the 1st Workshop on Healthcare AI and COVID-19, ICML 2022*, volume 184 of *Proceedings of Machine Learning Research*, pages 117–126. PMLR, 22 Jul 2022. URL `https://proceedings.mlr.press/v184/raza22a.html`.

[47] E. Hatef, M. Rouhizadeh, C. Nau, F. Xie, C. Rouillard, M. Abu-Nasser, A. Padilla, L.J. Lyons, H. Kharrazi, J.P. Weiner, and D. Roblin. Development and assessment of a natural language processing model to identify residential instability in electronic health records' unstructured data: A comparison of 3 integrated healthcare delivery systems. *JAMIA Open*, 5(1), 2022. doi:10.1093/jamiaopen/ooac006. Publisher: Oxford University Press.

[48] Christopher J. Rouillard, Mahmoud A. Nasser, Haihong Hu, and Douglas W. Roblin. Evaluation of a Natural Language Processing Approach to Identify Social Determinants of Health in Electronic Health Records in a Diverse Community Cohort. *Medical Care*, 60(3):248–255, March 2022. ISSN 1537-1948. doi:10.1097/MLR.0000000000001683.

[49] CA Bejan, M Ripperger, D Wilimitis, R Ahmed, J Kang, K Robinson, TJ Morley, DM Ruderfer, and CG Walsh. Improving ascertainment of suicidal ideation and suicide attempt with natural language processing. *SCIENTIFIC REPORTS*, 12(1), 2022. doi:10.1038/s41598-022-19358-3.

[50] S Boch, SA Hussain, S Bambach, C DeShetler, D Chisolm, and S Linwood. Locating Youth Exposed to Parental Justice Involvement in the Electronic Health Record: Development of a Natural Language Processing Model. *JMIR PEDIATRICS AND PARENTING*, 5(1), 2022.

doi:10.2196/33614.
[51] D.A. Dorr, A.R. Quiñones, T. King, M.Y. Wei, K. White, and C.A. Bejan. Prediction of Future Health Care Utilization Through Note-extracted Psychosocial Factors. *Med. Care*, 60(8):570–578, 2022. doi:10.1097/MLR.0000000000001742. Publisher: Lippincott Williams and Wilkins.
[52] S. Stewart De Ramirez, J. Shallat, K. Mcclure, R. Foulger, and L. Barenblat. Screening for Social Determinants of Health: Active and Passive Information Retrieval Methods. *Popul. Heath. Manage.*, 25(6):781–788, 2022. doi:10.1089/pop.2022.0228. Publisher: Mary Ann Liebert Inc.
[53] A. Teng and A. Wilcox. Simplified data science approach to extract social and behavioural determinants: A retrospective chart review. *BMJ Open*, 12(1), 2022. doi:10.1136/bmjopen-2020-048397. Publisher: BMJ Publishing Group.
[54] X. Zhao and A. Rios. A marker-based neural network system for extracting social determinants of health. *J Am Med Inform Assoc*, 30(8):1398–1407, 2023. doi:10.1093/jamia/ocad041. Publisher: NLM (Medline).
[55] S. Wang, Y. Dang, Z. Sun, Y. Ding, J. Pathak, C. Tao, Y. Xiao, and Y. Peng. An NLP approach to identify SDoH-related circumstance and suicide crisis from death investigation narratives. *J Am Med Inform Assoc*, 30(8):1408–1417, 2023. doi:10.1093/jamia/ocad068. Publisher: NLM (Medline).
[56] Geoffrey M Gray, Ayah Zirikly, Luis M Ahumada, Masoud Rouhizadeh, Thomas Richards, Christopher Kitchen, Iman Foroughmand, and Elham Hatef. Application of natural language processing to identify social needs from patient medical notes: development and assessment of a scalable, performant, and rule-based model in an integrated healthcare delivery system. *JAMIA Open*, 6(4):ooad085, December 2023. ISSN 2574-2531. doi:10.1093/jamiaopen/ooad085.
[57] A.B. Chapman, K. Cordasco, S. Chassman, T. Panadero, D. Agans, N. Jackson, K. Clair, R. Nelson, A.E. Montgomery, J. Tsai, E. Finley, and S. Gabrielian. Assessing longitudinal housing status using Electronic Health Record data: a comparison of natural language processing, structured data, and patient-reported history. *Frontier. Artif. Intell.*, 6, 2023. doi:10.3389/frai.2023.1187501. Publisher: Frontiers Media S.A.
[58] A. Mitra, R. Pradhan, R.D. Melamed, K. Chen, D.C. Hoaglin, K.L. Tucker, J.I. Reisman, Z. Yang, W. Liu, J. Tsai, and H. Yu. Associations between Natural Language Processing-Enriched Social Determinants of Health and Suicide Death among US Veterans. *JAMA Netw. Open*, 6 (3):E233079, 2023. doi:10.1001/jamanetworkopen.2023.3079. Publisher: American Medical Association.
[59] Chandreen Liyanage, Muskan Garg, Vijay Mago, and Sunghwan Sohn. Augmenting Reddit Posts to Determine Wellness Dimensions impacting Mental Health. pages 306–312. Association for Computational Linguistics, 2023. doi:10.18653/v1/2023.bionlp-1.27.
[60] Z. Yao, J. Tsai, W. Liu, D.A. Levy, E. Druhl, J.I. Reisman, and H. Yu. Automated identification of eviction status from electronic health record notes. *J Am Med Inform Assoc*, 30(8):1429–1437, 2023. doi:10.1093/jamia/ocad081. Publisher: NLM (Medline).
[61] Dmytro S Lituiev, Benjamin Lacar, Sang Pak, Peter L Abramowitsch, Emilia H De Marchis, and Thomas A Peterson. Automatic extraction of social determinants of health from medical notes of chronic lower back pain patients. *Journal of the American Medical Informatics Association*, 30(8):1438–1447, August 2023. ISSN 1527-974X. doi:10.1093/jamia/ocad054.
[62] Xiaoyu Wang, Dipankar Gupta, Michael Killian, and Zhe He. Benchmarking Transformer-Based Models for Identifying Social Determinants of Health in Clinical Notes. *Proceedings. IEEE International Conference on Healthcare Informatics*, 2023:570–574, June 2023. ISSN 2575-2626. doi:10.1109/ichi57859.2023.00102.
[63] C. Peng, X. Yang, Z. Yu, J. Bian, W.R. Hogan, and Y. Wu. Clinical concept and relation extraction using prompt-based machine reading comprehension. *J. Am. Med. Informatics Assoc.*, 30(9):1486–1493, 2023. doi:10.1093/jamia/ocad107. Publisher: Oxford University Press.
[64] R. Sajdeya, M.T. Mardini, P.J. Tighe, R.L. Ison, C. Bai, S. Jugl, G. Hanzhi, K. Zandbiglari, F.I. Adiba, A.G. Winterstein, T.A. Pearson, R.L. Cook, and M. Rouhizadeh. Developing and validating a natural language processing algorithm to extract preoperative cannabis use status documentation from unstructured narrative clinical notes. *J Am Med Inform Assoc*, 30(8):1418–1428, 2023. doi:10.1093/jamia/ocad080. Publisher: NLM (Medline).
[65] K. Lee, S. Han, and H.S. Suh. Early impact of COVID-19 social distancing on social determinants of health and their effects on mental health and quality of life of Korean undergraduate students. *Front. Public Health*, 11, 2023. doi:10.3389/fpubh.2023.1197143. Publisher: Frontiers Media SA.
[66] Ronald C. Kessler, Mark S. Bauer, Todd M. Bishop, Robert M. Bossarte, Victor M. Castro, Olga V. Demler, Sarah M. Gildea, Joseph L. Goulet, Andrew J. King, Chris J. Kennedy, Sara J. Landes, Howard Liu, Alex Luedtke, Patrick Mair, Brian P. Marx, Matthew K. Nock, Maria V. Petukhova, Wilfred R. Pigeon, Nancy A. Sampson, Jordan W. Smoller, Aletha Miller, Gretchen Haas, Jeffrey Benware, John Bradley, Richard R. Owen, Samuel House, Snezana Urosevic, and Lauren M. Weinstock. Evaluation of a model to target high-risk psychiatric inpatients for an intensive postdischarge suicide prevention intervention. *JAMA Psychiatry*, 80(3):230–240, 2023. doi:10.1001/jamapsychiatry.2022.4634.
[67] D.R. Harris, N. Anthony, D. Quesinberry, and C. Delcher. Evidence of housing instability identified by addresses, clinical notes, and diagnostic codes in a real-world population with substance use disorders. *J. Clin. Transl. Sci.*, 7(1), 2023. doi:10.1017/cts.2023.626. Publisher: Cambridge University Press.
[68] R. Richie, V.M. Ruiz, S. Han, L. Shi, and F.R. Tsui. Extracting social determinants of health events with transformer-based multitask, multilabel named entity recognition. *J Am Med Inform Assoc*, 30(8):1379–1388, 2023. doi:10.1093/jamia/ocad046. Publisher: NLM (Medline).
[69] B. Romanowski, A. Ben Abacha, and Y. Fan. Extracting social determinants of health from clinical note text with classification and sequence-to-sequence approaches. *J Am Med Inform Assoc*, 30(8):1448–1455, 2023. doi:10.1093/jamia/ocad071. Publisher: NLM (Medline).
[70] P.R. Shafer, A. Davis, and J.A. Clark. Finding social need-les in a haystack: ascertaining social needs of Medicare patients recorded in the notes of care managers. *BMC Health Serv. Res.*, 23(1), 2023. doi:10.1186/s12913-023-10446-2. Publisher: BioMed Central Ltd.
[71] T. Magoc, K.S. Allen, C. McDonnell, J.-P. Russo, J. Cummins, J.R. Vest, and C.A. Harle. Generalizability and portability of natural language processing system to extract individual social risk factors. *Int. J. Med. Informatics*, 177, 2023. doi:10.1016/j.ijmedinf.2023.105115. Publisher: Elsevier Ireland Ltd.
[72] U. Ravichandran, D. Jungst, and E. Kwan. Implementing an NLP Tool to Address SDOH Needs. pages 522–524. Institute of Electrical and Electronics Engineers Inc., 2023. doi:10.1109/ICHI57859.2023.00091.
[73] Jianfeng Zhu, Neha Yalamanchi, Ruoming Jin, Deric R. Kenne, and NhatHai Phan. Investigating COVID-19's impact on mental health: Trend and thematic analysis of Reddit users' discourse. *Journal of Medical Internet Research*, 25, 2023. doi:10.2196/46867.
[74] K. Lybarger, N.J. Dobbins, R. Long, A. Singh, P. Wedgeworth, Ö. Uzuner, and M. Yetisgen. Leveraging natural language processing to augment structured social determinants of health data in the electronic health record. *J Am Med Inform Assoc*, 30(8):1389–1397, 2023. doi:10.1093/jamia/ocad073. Publisher: NLM (Medline).
[75] Wenbo Wu, Kaes J. Holkeboer, Temidun O. Kolawole, Lorrie Carbone, and Elham Mahmoudi. Natural language processing to identify social determinants of health in Alzheimer's disease and related dementia from electronic health records. *Health Services Research*, 58(6):1292–1302, 2023. ISSN 1475-6773. doi:10.1111/1475-6773.14210.
[76] K.S. Allen, D.R. Hood, J. Cummins, S. Kasturi, E.A. Mendonca, and J.R. Vest. Natural language processing-driven state machines to extract social factors from unstructured clinical documentation. *JAMIA Open*, 6(2), 2023. doi:10.1093/jamiaopen/ooad024. Publisher: Oxford University Press.
[77] J.R. Vest and O. Mazurenko. Non-response Bias in Social Risk Factor Screening Among Adult Emergency Department Patients. *J. Med. Syst.*, 47(1), 2023. doi:10.1007/s10916-023-01975-8. Publisher: Springer.
[78] C.J. Cascalheira, S. Chapagain, R.E. Flinn, Y. Zhao, S.F. Boubrahimi, D. Klooster, A. Gonzalez, E.M. Lund, D. Laprade, J.R. Scheer, and S.M. Hamdi. Predicting Linguistically Sophisticated Social Determinants of Health Disparities with Neural Networks: The Case of LGBTQ+ Minority Stress. pages 1314–1321. Institute of Electrical and Electronics Engineers Inc., 2023. doi:10.1109/BigData59044.2023.10386882.
[79] G.K. Ramachandran, Y. Fu, B. Han, K. Lybarger, N.J. Dobbins, Ö. Uzuner, and M. Yetisgen. Prompt-based Extraction of Social Determinants of Health Using Few-shot Learning. pages 385–393. Association for Computational Linguistics (ACL), 2023. doi:10.18653/v1/2023.clinicalnlp-1.41.
[80] A.D. Lelkes, E. Loreaux, T. Schuster, M.-J. Chen, and A. Rajkomar. SDOH-NLI: a Dataset for Inferring Social Determinants of Health from Clinical Notes. pages 4789–4798. Association for Computational Linguistics (ACL), 2023. doi:10.18653/v1/2023.findings-emnlp.317.
[81] S. Mehta, C.R. Lyles, A.D. Rubinsky, K.E. Kemper, J. Auerbach, U. Sarkar, L. Gottlieb, and W. Brown. Social Determinants of Health Documentation in Structured and Unstructured Clinical Data of Patients With Diabetes: Comparative Analysis. *JMIR Med. Inform.*, 11, 2023. doi:10.2196/46159. Publisher: JMIR Publications Inc.
[82] Susan J. Burnett, Rachel Stemerman, Johanna C. Innes, Maria C. Kaisler, Remle P. Crowe, and Brian M. Clemency. Social Determinants of Health in EMS Records: A Mixed-methods Analysis Using Natural Language Processing and Qualitative Content Analysis. *Western Journal of Emergency Medicine: Integrating Emergency Care with Population Health*, 24(5), 2023. ISSN 1936-900X. doi:10.5811/westjem.59070.
[83] M. Hobensack, J. Song, S. Oh, L. Evans, A. Davoudi, K.H. Bowles, M.V. McDonald, Y. Barrón, S. Sridharan, A.S. Wallace, and M. Topaz. Social Risk Factors are Associated with Risk for Hospitalization in Home Health Care: A Natural Language Processing Study. *J. Am. Med. Dir. Assoc.*, 24(12):1874–1880.e4, 2023. doi:10.1016/j.jamda.2023.06.031. Publisher: Elsevier Inc.
[84] K. Lybarger, M. Yetisgen, and Ö. Uzuner. The 2022 n2c2/UW shared task on extracting social determinants of health. *J Am Med Inform Assoc*, 30(8):1367–1378, 2023. doi:10.1093/jamia/ocad012. Publisher: NLM (Medline).

[85] Tao Wang, David Codling, Dinesh Bhugra, Yamiko Msosa, Matthew Broadbent, Rashmi Patel, Angus Roberts, Philip McGuire, Robert Stewart, Richard Dobson, and Robert Harland. Unraveling ethnic disparities in antipsychotic prescribing among patients with psychosis: A retrospective cohort study based on electronic clinical records. *Schizophrenia Research*, 260:168–179, 2023. doi:10.1016/j.schres.2023.08.024.

[86] Briauna Lowery, Salvatore D'Acunto, Remle P. Crowe, and Jennifer N. Fishe. Using Natural Language Processing to Examine Social Determinants of Health in Prehospital Pediatric Encounters and Associations with EMS Transport Decisions. *Prehospital Emergency Care*, 27 (2):246–251, February 2023. ISSN 1090-3127. doi:10.1080/10903127.2022.2072984.

[87] F. Xie, S. Wang, L. Viveros, A. Rich, H.Q. Nguyen, A. Padilla, L. Lyons, and C.L. Nau. Using natural language processing to identify the status of homelessness and housing instability among serious illness patients from clinical notes in an integrated healthcare system. *JAMIA Open*, 6(3), 2023. doi:10.1093/jamiaopen/ooad082. Publisher: Oxford University Press.

[88] A.B. Chapman, D.O. Scharfstein, A.E. Montgomery, T. Byrne, Y. Suo, A. Effiong, T. Velasquez, W. Pettey, and R.E. Nelson. Using natural language processing to study homelessness longitudinally with electronic health record data subject to irregular observations. *AMIA Annu Symp Proc*, 2023:894–903, 2023. PMID: 38222404.

[89] Bhate NJ, Mittal A, He Z, and Luo X. Zero-shot Learning with Minimum Instruction to Extract Social Determinants and Family History from Clinical Notes using GPT Model. *Proc IEEE Int Conf Big Data*, 2023:1476–1480, 2023. doi:10.1109/BigData59044.2023.10386811. Place: United States.

[90] Fatemeh Shah-Mohammadi and Joseph Finkelstein. AI-Powered Social Determinants of Health Extraction from Patient Records: A GPT-Based Investigation. In *2024 IEEE First International Conference on Artificial Intelligence for Medicine, Health and Care (AIMHC)*, pages 109–112, February 2024. doi:10.1109/AIMHC59811.2024.00028.

[91] Z. Hu, Y. Zhang, R. Rossi, T. Yu, S. Kim, and S. Pan. Are Large Language Models Capable of Causal Reasoning for Sensing Data Analysis? pages 24–29. Association for Computing Machinery, Inc, 2024. doi:10.1145/3662006.3662064.

[92] MT Chandler, TR Cai, L Santacroce, S Ulysse, KP Liao, and CH Feldman. Classifying Individuals With Rheumatic Conditions as Financially Insecure Using Electronic Health Record Data and Natural Language Processing: Algorithm Derivation and Validation. *ACR OPEN RHEUMATOLOGY*, 2024. doi:10.1002/acr2.11675.

[93] O. Miranda, P. Fan, X. Qi, H. Wang, M.D. Brannock, T.R. Kosten, N.D. Ryan, L. Kirisci, and L. Wang. DeepBiomarker2: Prediction of Alcohol and Substance Use Disorder Risk in Post-Traumatic Stress Disorder Patients Using Electronic Medical Records and Multiple Social Determinants of Health. *J. Pers. Med.*, 14(1), 2024. doi:10.3390/jpm14010094. Publisher: Multidisciplinary Digital Publishing Institute (MDPI).

[94] K. Xie, W.K.S. Ojemann, R.S. Gallagher, R.T. Shinohara, A. Lucas, C.E. Hill, R.H. Hamilton, K.B. Johnson, D. Roth, B. Litt, and C.A. Ellis. Disparities in seizure outcomes revealed by large language models. *J. Am. Med. Informatics Assoc.*, 31(6):1348–1355, 2024. doi:10.1093/jamia/ocae047. Publisher: Oxford University Press.

[95] Lulu Wang, Yu Guo, Xuechen Yin, Yuhao Wang, and Ruipeng Tong. Exploring the determinants of health-promoting behaviors among miners: A text mining and meta-analysis. *Applied Psychology: Health and Well-Being*, 16(1):3–24, 2024. doi:10.1111/aphw.12465.

[96] Yujuan Fu, Giridhar Kaushik Ramachandran, Nicholas J. Dobbins, Namu Park, Michael Leu, Abby R. Rosenberg, Kevin Lybarger, Fei Xia, Özlem Uzuner, and Meliha Yetisgen. Extracting Social Determinants of Health from Pediatric Patient Notes Using Large Language Models: Novel Corpus and Methods. In Nicoletta Calzolari, Min-Yen Kan, Veronique Hoste, Alessandro Lenci, Sakriani Sakti, and Nianwen Xue, editors, *Proceedings of the 2024 Joint International Conference on Computational Linguistics, Language Resources and Evaluation (LREC-COLING 2024)*, pages 7045–7056, Torino, Italia, May 2024. ELRA and ICCL. URL `https://aclanthology.org/2024.lrec-main.618`.

[97] C Peng, X Yang, AK Chen, ZH Yu, KE Smith, AB Costa, MG Flores, J Bian, and YH Wu. Generative large language models are all-purpose text analytics engines: text-to-text learning is all your need. *JOURNAL OF THE AMERICAN MEDICAL INFORMATICS ASSOCIATION*, 2024. doi:10.1093/jamia/ocae078.

[98] T. Huang, V. Socrates, A. Gilson, C. Safranek, L. Chi, E.A. Wang, L.B. Puglisi, C. Brandt, R.A. Taylor, and K. Wang. Identifying incarceration status in the electronic health record using large language models in emergency department settings. *J. Clin. Transl. Sci.*, 8(1), 2024. doi:10.1017/cts.2024.496. Publisher: Cambridge University Press.

[99] Z. Yu, C. Peng, X. Yang, C. Dang, P. Adekkanattu, B. Gopal Patra, Y. Peng, J. Pathak, D.L. Wilson, C.-Y. Chang, W.-H. Lo-Ciganic, T.J. George, W.R. Hogan, Y. Guo, J. Bian, and Y. Wu. Identifying social determinants of health from clinical narratives: A study of performance, documentation ratio, and potential bias. *J. Biomed. Informatics*, 153, 2024. doi:10.1016/j.jbi.2024.104642. Publisher: Academic Press Inc.

[100] Christopher Whitfield, Yang Liu, and Mohd Anwar. Impact of COVID-19 Pandemic on Social Determinants of Health Issues of Marginalized Black and Asian Communities: A Social Media Analysis Empowered by Natural Language Processing. *Journal of Racial and Ethnic Health Disparities*, April 2024. ISSN 2196-8837. doi:10.1007/s40615-024-01996-0.

[101] L. Cybulski, N. Chilman, A. Jewell, M. Dewey, R. Hildersley, C. Morgan, R. Huck, M. Hotopf, R. Stewart, M. Pritchard, M. Wuerth, and J. Das-Munshi. Improving our understanding of the social determinants of mental health: a data linkage study of mental health records and the 2011 UK census. *BMJ Open*, 14(1), 2024. doi:10.1136/bmjopen-2023-073582. Publisher: BMJ Publishing Group.

[102] Purpura A, Mulligan N, Kartoun U, Koski E, Anand V, and Bettencourt-Silva J. Investigating Cross-Domain Binary Relation Classification in Biomedical Natural Language Processing. *AMIA Jt Summits Transl Sci Proc*, 2024:384–390, 2024. PMID: 38827064.

[103] M. Guevara, S. Chen, S. Thomas, T.L. Chaunzwa, I. Franco, B.H. Kann, S. Moningi, J.M. Qian, M. Goldstein, S. Harper, H.J.W.L. Aerts, P.J. Catalano, G.K. Savova, R.H. Mak, and D.S. Bitterman. Large language models to identify social determinants of health in electronic health records. *npj Digit. Med.*, 7(1), 2024. doi:10.1038/s41746-023-00970-0. Publisher: Nature Research.

[104] C. Peng, X. Yang, K.E. Smith, Z. Yu, A. Chen, J. Bian, and Y. Wu. Model tuning or prompt Tuning? a study of large language models for clinical concept and relation extraction. *J. Biomed. Informatics*, 153, 2024. doi:10.1016/j.jbi.2024.104630. Publisher: Academic Press Inc.

[105] K. Bhanot, J.S. Erickson, and K.P. Bennett. MortalityMinder: Visualization and AI Interpretations of Social Determinants of Premature Mortality in the United States. *Information*, 15(5), 2024. doi:10.3390/info15050254. Publisher: Multidisciplinary Digital Publishing Institute (MDPI).

[106] S. Kwon, X. Wang, W. Liu, E. Druhl, M.L. Sung, J.I. Reisman, W. Li, R.D. Kerns, W. Becker, and H. Yu. ODD: A Benchmark Dataset for the Natural Language Processing Based Opioid Related Aberrant Behavior Detection. volume 1, pages 4338–4359. Association for Computational Linguistics (ACL), 2024. doi:10.18653/v1/2024.naacl-long.244.

[107] Evan V. Goldstein, Elise V. Bailey, and Fernando A. Wilson. Poverty and suicidal ideation among Hispanic mental health care patients leading up to the COVID-19 pandemic. *Hispanic Health Care International*, 22(1):6–10, 2024. doi:10.1177/15404153231181110.

[108] M Mahbub, I Goethert, I Danciu, K Knight, S Srinivasan, S Tamang, K Rozenberg-Ben-Dror, H Solares, S Martins, J Trafton, E Begoli, and GD Peterson. Question-answering system extracts information on injection drug use from clinical notes. *COMMUNICATIONS MEDICINE*, 4 (1), 2024. doi:10.1038/s43856-024-00470-6.

[109] C Suda-King, L Winch, JM Tucker, AD Zuehlke, C Hunter, and JM Simmons. Representation of Social Determinants of Health terminology in medical subject headings: impact of added terms. *JOURNAL OF THE AMERICAN MEDICAL INFORMATICS ASSOCIATION*, 2024. doi:10.1093/jamia/ocae191.

[110] D.R. Harris, S. Fu, A. Wen, A. Corbeau, D. Henderson, J. Hilsman, D. Oniani, and Y. Wang. The ENACT Network is Acting on Housing Instability and the Unhoused Using the Open Health Natural Language Processing Toolkit. *J. Clin. Transl. Sci.*, 2024. doi:10.1017/cts.2024.543. Publisher: Cambridge University Press.

[111] Shenghuan Sun, Travis Zack, Christopher Y K Williams, Madhumita Sushil, and Atul J Butte. Topic modeling on clinical social work notes for exploring social determinants of health factors. *JAMIA Open*, 7(1):ooad112, April 2024. ISSN 2574-2531. doi:10.1093/jamiaopen/ooad112.

[112] Natasha Chilman, Dionne Laporte, Sarah Dorrington, Stephani L. Hatch, Craig Morgan, Celestin Okoroji, Robert Stewart, and Jayati Das-Munshi. Understanding social and clinical associations with unemployment for people with schizophrenia and bipolar disorders: Large-scale health records study. *Social Psychiatry and Psychiatric Epidemiology: The International Journal for Research in Social and Genetic Epidemiology and Mental Health Services*, 2024. doi:10.1007/s00127-024-02620-6.

[113] M. Ashayeri and N. Abbasabadi. Unraveling energy justice in NYC urban buildings through social media sentiment analysis and transformer deep learning. *Energy Build.*, 306, 2024. doi:10.1016/j.enbuild.2024.113914. Publisher: Elsevier Ltd.

[114] G. Savcisens, T. Eliassi-Rad, L.K. Hansen, L.H. Mortensen, L. Lilleholt, A. Rogers, I. Zettler, and S. Lehmann. Using sequences of life-events to predict human lives. *Nat. Comput. Sci.*, 4(1):43–56, 2024. doi:10.1038/s43588-023-00573-5. Publisher: Springer Nature.

[115] Krishnamoorthy R, Nagarajan V, Pour H, Shashikumar SP, Boussina A, Farcas E, Nemati S, and Josef CS. Voice-Enabled Response Analysis Agent (VERAA): Leveraging Large Language Models to Map Voice Responses in SDoH Survey. *AMIA Jt Summits Transl Sci Proc*, 2024: 258–265, 2024. PMID: 38827075.

[116] X. Luo, F.M. Tahabi, T. Marc, L.A. Haunert, and S. Storey. Zero-shot learning to extract assessment criteria and medical services from the preventive healthcare guidelines using large language models. *J. Am. Med. Informatics Assoc.*, 31(8):1743–1753, 2024. doi:10.1093/jamia/ocae145. Publisher: Oxford University Press.

[117] Gabriel RA, Litake O, Simpson S, Burton BN, Waterman RS, and Macias AA. On the development and validation of large language model-based classifiers for identifying social determinants of health. *Proc Natl Acad Sci U S A*, 121(39):e2320716121, 2024. doi:10.1073/pnas.2320716121. Place: United States.

[118] L. Gong, A. Shor, A. Zhang, and K. Jha. Context-Specific Feature Augmentation for Improving Social Determinants of Health Extraction. pages 1736–1745, 2024. doi:10.1109/BigData62323.2024.10825225.

[119] Gong L, Bresnick J, Zhang A, Wu C, and Jha K. Boosting Social Determinants of Health Extraction with Semantic Knowledge Augmented Large Language Model. *AMIA Annu Symp Proc*, 2024:453–462, 2024. PMID: 40417469.

[120] Martin EA, D'Souza AG, Saini V, Tang K, Quan H, and Eastwood CA. Extracting social determinants of health from inpatient electronic medical records using natural language processing. *J Epidemiol Popul Health*, 72(6):202791, 2024. doi:10.1016/j.jeph.2024.202791. Place: France.

[121] Moon S, Wu Y, Doughty JB, Wieland ML, Philpot LM, Fan JW, and Njeru JW. Automated Identification of Patients' Unmet Social Needs in Clinical Text Using Natural Language Processing. *Mayo Clin Proc Digit Health*, 2(3):411–420, 2024. doi:10.1016/j.mcpdig.2024.06.008. Place: Netherlands.

[122] Ralevski A, Taiyab N, Nossal M, Mico L, Piekos S, and Hadlock J. Using Large Language Models to Abstract Complex Social Determinants of Health From Original and Deidentified Medical Notes: Development and Validation Study. *J Med Internet Res*, 26:e63445, 2024. doi:10.2196/63445. Place: Canada.

[123] D. Roosan, J. Chok, Y. Li, and T. Khou. Utilizing Quantum Computing-based Large Language Transformer Models to Identify Social Determinants of Health from Electronic Health Records. 2024. doi:10.1109/ICECET61485.2024.10698600.

[124] Roy S, Morrell S, Zhao L, and Homayouni R. Large-scale identification of social and behavioral determinants of health from clinical notes: comparison of Latent Semantic Indexing and Generative Pretrained Transformer (GPT) models. *BMC Med Inform Decis Mak*, 24(1):296, 2024. doi:10.1186/s12911-024-02705-x. Place: England.

[125] Chapman AB, Panadero T, Dalrymple R, Cohen A, Kamdar N, Pethani F, Kalvesmaki A, Nelson RE, and Butler J. Studying Veteran food insecurity longitudinally using electronic health record data and natural language processing. *AMIA Jt Summits Transl Sci Proc*, 2025:124–133, 2025. PMID: 40502216.

[126] Chen Z, Lasserre P, Lin A, and Rajapakshe R. Extraction of Social Determinants of Health From Electronic Health Records Using Natural Language Processing. *JCO Clin Cancer Inform*, 9:e2400317, 2025. doi:10.1200/CCI-24-00317. Place: United States.

[127] Consoli B, Wang H, Wu X, Wang S, Zhao X, Wang Y, Rousseau J, Hartvigsen T, Shen L, Wu H, Peng Y, Long Q, Chen T, and Ding Y. SDoH-GPT: using large language models to extract social determinants of health. *J Am Med Inform Assoc*, 2025. doi:10.1093/jamia/ocaf094. Place: England.

[128] Gu B, Shao V, Liao Z, Carducci V, Brufau SR, Yang J, and Desai RJ. Scalable information extraction from free text electronic health records using large language models. *BMC Med Res Methodol*, 25(1):23, 2025. doi:10.1186/s12874-025-02470-z. Place: England.

[129] Gu Z, He L, Naeem A, Chan PM, Mohamed A, Khalil H, Guo Y, Huang J, Villanueva-Miranda I, Ding Y, Shi W, Dupre ME, Xiao G, Peterson ED, Xie Y, Navar AM, and Yang DM. SBDH-Reader: a large language model-powered method for extracting social and behavioral determinants of health from clinical notes. *J Am Med Inform Assoc*, 32(10):1570–1580, 2025. doi:10.1093/jamia/ocaf124. Place: England.

[130] Keloth VK, Selek S, Chen Q, Gilman C, Fu S, Dang Y, Chen X, Hu X, Zhou Y, He H, Fan JW, Wang K, Brandt C, Tao C, Liu H, and Xu H. Social determinants of health extraction from clinical notes across institutions using large language models. *NPJ Digit Med*, 8(1):287, 2025. doi:10.1038/s41746-025-01645-8. Place: England.

[131] Kim MH, Miramontes S, Mehta S, Schwartz GL, Kim YJ, Yang Y, Hill-Jarrett TG, Cevallos N, Chen R, Glymour MM, Ferguson EL, Zimmerman SC, Choi M, and Sims KD. Extracting Housing and Food Insecurity Information From Clinical Notes Using cTAKES. *Health Serv Res*, 60 Suppl 3(Suppl 3):e14440, 2025. doi:10.1111/1475-6773.14440. Place: United States.

[132] Patra BG, Lepow LA, Kasi Reddy Jagadeesh Kumar P, Vekaria V, Sharma MM, Adekkanattu P, Fennessy B, Hynes G, Landi I, Sanchez-Ruiz JA, Ryu E, Biernacka JM, Nadkarni GN, Talati A, Weissman M, Olfson M, Mann JJ, Zhang Y, Charney AW, and Pathak J. Extracting social support and social isolation information from clinical psychiatry notes: comparing a rule-based natural language processing system and a large language model. *J Am Med Inform Assoc*, 32(1):218–226, 2025. doi:10.1093/jamia/ocae260. Place: England.

[133] Peng C, Yu Z, Smith KE, Lo-Ciganic WH, Bian J, and Wu Y. Enhancing Cross-Domain Generalizability in Social Determinants of Health Extraction with Prompt-Tuning Large Language Models. *AMIA Jt Summits Transl Sci Proc*, 2025:432–440, 2025. PMID: 40502248.

[134] Fardin Ahsan Sakib, Ziwei Zhu, Karen Trister Grace, Meliha Yetisgen, and Ozlem Uzuner. Spurious Correlations and Beyond: Understanding and Mitigating Shortcut Learning in SDOH Extraction with Large Language Models. pages 1097–1106. Association for Computational Linguistics, 2025. doi:10.18653/v1/2025.acl-short.86.

[135] Scherbakov D, Heider PM, Wehbe R, Alekseyenko AV, Lenert LA, and Obeid JS. Using large language models for extracting stressful life events to assess their impact on preventive colon cancer screening adherence. *BMC Public Health*, 25(1):12, 2025. doi:10.1186/s12889-024-21123-2. Place: England.

[136] Shang T, Yang S, He W, Zhai T, Li D, Hou B, Chen T, Moore JH, Ritchie MD, and Shen L. Leveraging Social Determinants of Health in Alzheimer's Research Using LLM-Augmented Literature Mining and Knowledge Graphs. *AMIA Jt Summits Transl Sci Proc*, 2025:491–500, 2025. PMID: 40502260.

[137] Shao M, Kang Y, Hu X, Kwak HG, Yang C, and Lu J. Mining Social Determinants of Health for Heart Failure Patient 30-Day Readmission via Large Language Model. *Stud Health Technol Inform*, 329:1902–1903, 2025. doi:10.3233/SHTI251272. Place: Netherlands.

[138] Soley N, Bentil M, Shah J, Rouhizadeh M, and Taylor CO. Unveiling social determinants of health impact on adverse pregnancy outcomes through natural language processing. *Sci Rep*, 15(1):29183, 2025. doi:10.1038/s41598-025-13542-x. Place: England.

[139] Wang S, Wei Y, Ma H, Lovitt M, Deng K, Meng Y, Xu Z, Zhang J, Xiao Y, Ding Y, Xu X, Ghosh J, and Peng Y. A multi-stage large language model framework for extracting suicide-related social determinants of health. *Commun Med (Lond)*, 5(1):404, 2025. doi:10.1038/s43856-025-01114-z. Place: England.

[140] Wang Y, Hilsman J, Li C, Morris M, Heider PM, Fu S, Kwak MJ, Wen A, Applegate JR, Wang L, Bernstam E, Liu H, Chang J, Harris DR, Corbeau A, Henderson D, Osborne J, Kennedy RE, Garduno-Rapp NE, Rousseau JF, Yan C, Chen Y, Patel MB, Murphy TJ, Malin BA, Park CM, Fan JW, Sohn S, Pagali S, Peng Y, Pathak A, Wu Y, Xia Z, Loguercio S, Reis SE, and Visweswaran S. Development and validation of natural language processing algorithms in the national ENACT network. *J Clin Transl Sci*, 9(1):e199, 2025. doi:10.1017/cts.2025.10116. Place: England.

[141] Wasser LM, Liang HW, Li C, Cassidy J, Tallapaneni P, Osterhoudt H, Wang Y, and Williams AM. Identifying Transportation Needs in Ophthalmology Clinic Notes Using Natural Language Processing: Retrospective, Cross-Sectional Study. *JMIR Med Inform*, 13:e69216, 2025. doi:10.2196/69216. Place: Canada.

[142] Zaribafzadeh H, Henson JB, Chan NW, Rogers U, Webster W, Schappe T, Li F, Matsouaka RA, Kirk AD, Henao R, and McElroy LM. Development of a natural language processing algorithm to extract social determinants of health from clinician notes. *Am J Transplant*, 25(6): 1306–1318, 2025. doi:10.1016/j.ajt.2025.02.019. Place: United States.
