# Supplementary material for: Applications of Natural Language Processing and Large Language Models for Social Determinants of Health: Systematic Review
Source: J Med Internet Res. 2026 Apr 28;28:e83793. doi: 10.2196/83793 (PMC13123760; doi:10.2196/83793)
Supplement: Checklist 1 [file jmir-v28-e83793-s002.pdf]

PRISMA Checklist

Table 1: PRISMA 2020 Checklist

Section and Topic	Item	Checklist Item	Item Location (Section name)
**TITLE**			
Title	1	Identify the report as a systematic review.	Title
**ABSTRACT**			
Abstract	2	See the PRISMA 2020 for Abstracts Checklist.	Abstract
**INTRODUCTION**			
Rationale	3	Describe the rationale for the review in the context of existing knowledge.	Introduction
Objectives	4	Provide an explicit statement of the objective(s) or question(s) the review addresses.	Introduction
**METHODS**			
Eligibility Criteria	5	Specify the inclusion and exclusion criteria for the review and how studies were grouped for the syntheses.	Eligibility Criteria
Information sources	6	Specify all databases, registers, websites, organisations, reference lists and other sources searched or consulted to identify studies. Specify the date when each source was last searched or consulted.	Database and Search Strategy
Search strategy	7	Present the full search strategies for all databases, registers and websites, including any filters and limits used.	Database and Search Strategy
Selection process	8	Specify the methods used to decide whether a study met the inclusion criteria, including how many reviewers screened each record, whether they worked independently, and details of automation tools used.	Selection process
Data collection process	9	Specify the methods used to collect data from reports, including how many reviewers collected data, whether they worked independently, and any processes for obtaining or confirming data from study investigators.	Data Extraction
Data items	10a	List and define all outcomes for which data were sought. Specify whether all results that were compatible with each outcome domain in each study were sought, and if not, the methods used to decide which results to collect.	Data Extraction
	10b	List and define all other variables for which data were sought (e.g. participant characteristics, funding sources). Describe any assumptions made about missing or unclear information.	
Study risk of bias assessment	11	Specify the methods used to assess risk of bias in the included studies, including the tools used, how many reviewers assessed each study, and whether they worked independently.	Data Extraction
Effect measures	12	Specify for each outcome the effect measure(s) (e.g. risk ratio, mean difference) used in the synthesis or presentation of results.	Data Extraction
Synthesis methods	13a	Describe the processes used to decide which studies were eligible for each synthesis.	Eligibility criteria
	13b	Describe any methods required to prepare the data for presentation or synthesis, such as handling of missing statistics or data conversions.	Data Extraction
	13c	Describe any methods used to tabulate or visually display results of individual studies and syntheses.	Data Extraction
	13d	Describe any methods used to synthesize results and provide a rationale for the choice(s). If meta-analysis was performed, describe the model(s), method(s) to identify heterogeneity, and software used.	Meta analysis not performed
	13e	Describe any methods used to explore possible causes of heterogeneity among study results.	Registration and Protocol
	13f	Describe any sensitivity analyses conducted to assess robustness of the synthesized results.	Registration and Protocol
Reporting bias assessment	14	Describe any methods used to assess risk of bias due to missing results in a synthesis (e.g. arising from reporting biases).	Registration and Protocol
Certainty assessment	15	Describe any methods used to assess certainty in the body of evidence for an outcome.	Registration and Protocol

Table 2: PRISMA 2020 Checklist, Cont'd.

Section and Topic	Item	Checklist Item	Item Location (Section name)
**RESULTS**			
Study selection	16a	Describe the results of the search and selection process, from the number of records identified in the search to the number of studies included in the review, ideally using a flow diagram.	Study selection
	16b	Cite studies that might appear to meet the inclusion criteria, but which were excluded, and explain why they were excluded.	and characteristics
Study Characteristics	17	Cite each included study and present its characteristics.	Study selection & characteristics
Risk of bias in studies	18	Present assessments of risk of bias for each included study.	Results and Table 2
Results of individual studies	19	For all outcomes, present, for each study: (a) summary statistics for each group (where appropriate) and (b) an effect estimate and its precision (e.g. confidence/credible interval), ideally using structured tables or plots.	NLP methods and performance
Results of syntheses	20a	For each synthesis, briefly summarize the characteristics and risk of bias among contributing studies.	Data sources and characteristics
	20b	Present results of all statistical syntheses conducted. If meta-analysis was done, present for each the summary estimate and its precision (e.g. confidence/credible interval) and measures of statistical heterogeneity. If comparing groups, describe the direction of the effect.	Not a meta analysis
	20c	Present results of all investigations of possible causes of heterogeneity among study results.	Study Selection and Characteristics
	20d	Present results of all sensitivity analyses conducted to assess the robustness of the synthesized results.	Study Selection and Characteristics
Reporting biases	21	Present assessments of risk of bias due to missing results (arising from reporting biases) for each synthesis assessed.	Study Selection and Characteristics
Certainty of evidence	22	Present assessments of certainty (or confidence) in the body of evidence for each outcome assessed.	Study Selection and Characteristics
**DISCUSSION**			
Discussion	23a	Provide a general interpretation of the results in the context of other evidence.	Discussion
	23b	Discuss any limitations of the evidence included in the review.	Discussion
	23c	Discuss any limitations of the review processes used.	Limitations
	23d	Discuss implications of the results for practice, policy, and future research.	Future research directions
**OTHER INFORMATION**			
Registration and protocol	24a	Provide registration information for the review, including register name and registration number, or state that the review was not registered.	Registration
	24b	Indicate where the review protocol can be accessed, or state that a protocol was not prepared.	
	24c	Describe and explain any amendments to the information provided at registration or in the protocol.	and protocol
Support	25	Describe sources of financial or non-financial support for the review, and the role of the funders or sponsors in the review.	Funding
Competing interests	26	Declare any competing interests of review authors.	Competing interests
Availability of data, code, and other materials	27	Report which of the following are publicly available and where they can be found: template data collection forms; data extracted from included studies; data used for all analyses; analytic code; any other materials used in the review.	Data availability
